# Mutant p53 gain of function mediates cancer immune escape that is counteracted by APR-246

**DOI:** 10.1038/s41416-022-01971-8

**Published:** 2022-09-22

**Authors:** Xiaolei Zhou, Gema Sanz Santos, Yue Zhan, Mariana M. S. Oliveira, Shiva Rezaei, Madhurendra Singh, Sylvain Peuget, Lisa S. Westerberg, John Inge Johnsen, Galina Selivanova

**Affiliations:** 1 Department of Microbiology, Tumor and Cell Biology, Karolinska Institutet, 171 65 Stockholm, Sweden; 2 Department of Breast Surgery, The First Hospital of Jilin University, Changchun, China; 3 Department of Women’s and Children’s Health, Childhood Cancer Research Unit, Karolinska Institutet, 171 77 Stockholm, Sweden; 4 Department of Microbiology, Tumor and Cell Biology, Biomedicum C8, Karolinska Institutet, 171 65 Stockholm, Sweden

**Keywords:** Immunosurveillance, Targeted therapies

## Abstract

**Background:**

p53 mutants contribute to the chronic inflammatory tumour microenvironment (TME). In this study, we address the mechanism of how p53 mutants lead to chronic inflammation in tumours and how to transform it to restore cancer immune surveillance.

**Methods:**

Our analysis of RNA-seq data from The Cancer Genome Atlas Breast Invasive Carcinoma (TCGA-BRCA) project revealed that mutant p53 (mtp53) cancers correlated with chronic inflammation. We used cell-based assays and a mouse model to discover a novel gain of function of mtp53 and the effect of the mtp53 reactivating compound APR-246 on the anti-tumour immune response.

**Results:**

We found that tumour samples from patients with breast carcinoma carrying mtp53 showed elevated Interferon (IFN) signalling, Tumour Inflammation Signature (TIS) score and infiltration of CD8+ T cells compared to wild type p53 (wtp53) tumours. We showed that the expression of IFN and immune checkpoints were elevated in tumour cells in a mtp53-dependent manner, suggesting a novel gain of function. Restoration of wt function to mtp53 by APR-246 induced the expression of endogenous retroviruses, IFN signalling and repressed immune checkpoints. Moreover, APR-246 promoted CD4+ T cells infiltration and IFN signalling and prevented CD8+ T cells exhaustion within the TME in vivo.

**Conclusions:**

Breast carcinomas with mtp53 displayed enhanced inflammation. APR-246 boosted the interferon response or represses immune checkpoints in p53 mutant tumour cells, and restores cancer immune surveillance in vivo.

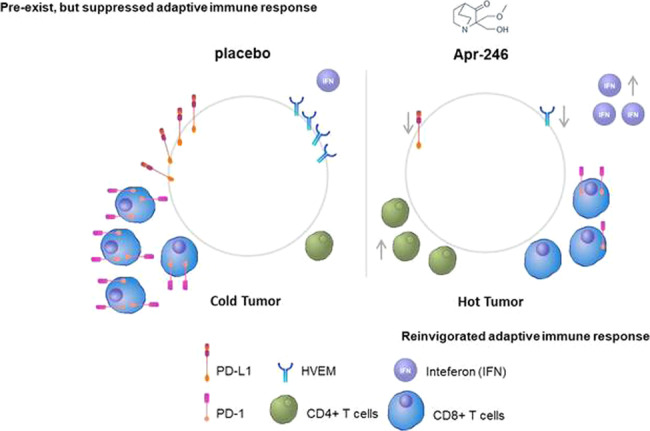

## Introduction

It is well documented that chronic inflammation can lead to cancer [1, 2]. During the past decade, tumour promoting inflammation has been identified as a new hallmark of cancer [3]. Numerous lines of evidence suggest that p53 is a strong suppressor of inflammation. Wild-type (wt) p53 inhibits the main regulator of inflammation, nuclear factor-κB (NF-κB), whose constitutive activation is believed to be the fundamental cause of chronic inflammation [4, 5]. Wtp53 also mediates the suppression of inflammatory cytokines [6] and regulation of T cell differentiation [7].

p53 is inactivated by mutation in almost half of all human cancers. In addition to loss of tumour suppression function, some p53 mutants acquire oncogenic properties, so called “gain of function” (GOF), which facilitate tumour progression. Several studies have shown that p53 mutants contribute to the inflammatory TME in several contexts, but the mechanisms of this phenomenon are not entirely clear (reviewed in ref. [8]). Therefore, it would be important to find out the molecular mechanisms of mtp53-mediated inflammatory phenotype and address the question whether restoration of wild type function to mtp53 can counteract mtp53-mediated immune evasion.

Interferons (IFNs) are a group of cytokines, including type I, II and III IFNs, which play a critical anti-cancer role. IFNs can induce tumour cells apoptosis and activate both innate and adaptive immune response against tumours [9]. Type II IFN is mainly expressed by immune cells, while type I IFN, including IFNα and IFNβ, and type III IFNs, comprising IFNλ1, IFNλ2/3 and IFNλ4, are expressed ubiquitously in human cells. Mtp53 suppresses IFNβ production via inhibiting the dsDNA sensing pathway TBK1-STING-IRF3 [10]. In addition, mtp53 can sustain tumour cells survival by suppression of IFNβ that is secreted by cancer-associated fibroblasts through inhibition of STAT1 phosphorylation [11].

Mtp53-reactivating compound APR-246 (commercial name eprenetapopt), identified by us [12], has been shown to suppress tumours not only via restoration of p53 tumour suppressor function, but also by other mechanisms, including induction of oxidative stress, endoplasmic reticulum (ER) stress and ferroptosis [13, 14]. APR-246 is currently being tested in several clinical trials ranging from phase I to phase III, either alone or in combination with other anti-cancer agents, in various types of cancer (clinicaltrials.gov). Recent studies showed that APR-246 in combination with azacitidine is well tolerated in *TP53* mutant MDS patients and has impressive clinical response rates in Phase Ib/II studies [15, 16].

Immune suppression is believed to be essential for tumour development and tumour progression (reviewed in refs. [17, 18]). Tumour cells can escape from immune surveillance through tumour intrinsic and/or tumour extrinsic mechanisms. One of the common tumour intrinsic ways to suppress CD8+ cytotoxic T lymphocytes (CTLs) is to upregulate negative immune checkpoint molecules on the cell surface including the most studied molecule PD-L1, and also several others, HVEM, PVR, PVRL2 and CD276 [19–22]. Whether and how mtp53 or APR-246 affects the expression of these immune checkpoint molecules is currently unclear.

We recently discovered that the pharmacological activation of wtp53 can induce anti-cancer IFN response in cancer cell lines, mouse models and patients via de-repression of ERVs and induction of dsRNA stress, as well as facilitate anti-tumour immune cells infiltration within TME in two mouse models [23]. Taking into account these results, we addressed the question, whether mtp53 is involved in the regulation of ERVs and IFNs expression and whether restoration of wtp53 function to mtp53 by APR-246 can boost the IFN response, regulate immune checkpoints and promote immune cell infiltration in vivo.

We found that the TME of breast cancer patients carrying mtp53 showed signs of inflammation, as exemplified by enhanced TIS score, enhanced IFN signalling, and CD8+ T cells infiltration. However, the adaptive immune response in mtp53 breast cancers is suppressed, as evidenced by elevated tumour intrinsic and extrinsic negative regulators of CD8+ T cells. Treatment of mtp53 cancer cells by APR-246 either boosts IFN response or inhibits the expression of negative immune checkpoints, depending on the mechanism of the tumour cell escape from immune surveillance. Moreover, APR-246 promoted the infiltration of CD4+ T cells and prevented the exhaustion of CD8+ T cells within TME in a mouse model of fibrosarcoma.

## Methods

### RNA extraction and reverse transcription

To extract RNA from total cell lysate and reverse transcribe RNA to cDNA, Aurum^TM^ Total RNA Mini Kit (Bio-Rad cat#7326820) and iScript^TM^ Reverse Transcription Supermix (Bio-Rad cat#1708841), respectively, was used according to the manufacture’s protocol. RNA concentration was quantified by nanodrop and 1 μg of RNA was used to synthesise cDNA.

### Real-time quantitative PCR (RT-qPCR)

To quantify relative gene expression at mRNA level, RT-qPCR was used. Five microlitres of reaction system including 2 μl cDNA, 0.5 μl of 5 μM primer mix and 2 μl SsoAdvanced Universal SYBR Green Supermix (Bio-Rad cat#1725275) was loaded in a 384 plate, which was next transferred to a CFX384 Touch™ Real-Time PCR machine. The running program used and data analysis are specified in Supplementary Method. Every experiment has been repeated three times (three biological replicates) and each biological replicate contains three technical repeats.

### Western blot

To quantify genes expression at protein level, immunoblot was used. Total protein was extracted by using RIPA buffer method and protein concentration was measured by either the Bradford or BCA method. Gels were casted manually ranging from 7.5 to 12.5% acrylamide concentration depending on the size of the target protein detected. In all, 20 μg to 40 μg total protein was used to load on casted gels and wet transfer was selected to transfer proteins on gels to nitrocellulose membrane. The detailed protocol and antibodies used can be found in Supplementary Method.

### Lentivirus- or siRNA-mediated p53 knockdown

To knockdown p53 in human cancer cell lines with mtp53, lentivirus containing human TP53 shRNA (sequence listed in Supplementary Table S2) was transduced. Lentivirus production and transduction procedure were performed as previously described [24]. The transduction efficiency was monitored by detection of GFP by immunofluorescence microscope and the knockdown efficiency of mtp53 was detected by western blot. Murine p53 was depleted by 10 nM siRNA SMARTPool targeting murine *TP53* (Catalogue ID:L-040642-00-0010, Dharmacon) with INTERFERin siRNA transfection reagent (Cat# 101000028, Polyplus).

### Animal experiments

Animal experiments were approved by the regional ethics committee for animal study (reference number: 13820-2019), and under the supervision of the Swedish Board of Agriculture and the Swedish Court. All animal experiments performed in this study were in line with national regulations (SFS 1988:534, SFS 1988:539 and SFS 1988:541).

To evaluate whether APR-246 can promote immune cell infiltration within the TME of mtp53 tumours, the MCO4 murine fibrosarcoma was injected subcutaneously into BALB/C mice as previously documented [25]. Mice were randomised into two experimental groups including group 1 to evaluate the tumour suppression effect of APR-246 and group 2 to evaluate how APR-246 affect immune cell infiltration within the TME. After the tumour size reached around 100 mm^3^ in group 1, 100 μl APR-246 in PBS was administered daily by i.p. injection at a dosage of 100 mg/kg mouse body weight for 2 weeks. Tumour size and mouse body weight were recorded every other day. In group 2, after tumour size reached around 300 mm^3^, 100 μl of APR-246 in PBS was administered daily by i.p. injection at a dosage of 100 mg/kg mouse body weight until day 4. Mice were sacrificed at day 5 and tumours were isolated from mice. Detailed protocol of single cell preparation, immunoprofiling and antibodies used could be found in Supplementary method.

### TCGA data analysis

Immune-related gene expression profiles in breast invasive carcinoma were downloaded from cBioPortal for Cancer Genomics (http://www.cbioportal.org/, BRCA, Firehose Legacy data set) [26]. Only those samples which contain at least 60% of tumour cells have been used for RNA-Seq. RNA-Seq V2 data expressed as mRNA z-scores with 2 threshold and the p53 mutational status data were downloaded for each patient sample and grouped into wild type in the absence of detectable p53 mutation, or mutant if there was a missense mutation or in frame deletion/insertion (wtp53 = 618, mtp53 = 258). Statistical significance was calculated using Mann–Whitney test (*p ≤ 0.05, **p ≤ 0.01, ***p ≤ 0.001, ****p ≤ 0.0001). Data were plotted using Graphpad Prism version 7 (GraphPad Software, San Diego, CA, USA).

Hierarchical clustering was computed and visualised using ComplexHeatmap R package [27].

HTSeq-Counts data of RNA-Seq in TCGA-BRCA project were downloaded from GDC Data Portal by using the GDC data transfer tool provided by TCGA website. The data were grouped into either wtp53 (690) or mtp53 (401). For specific gene expression, raw counts were removed if read counts was <10 in >80% of the samples. Analysis of differential gene expression between mtp53 samples and wtp53 samples was performed by DESeq2 R package. Gene list with log2 FoldChange >1 and adjusted *p* value < 0.05 was selected and KEGG enrichment analysis was performed by clusterProfiler R package. Figures were prepared using ggplot2 R package.

## Results

### Elevated TIS score and IFN signalling in breast cancer patients carrying mtp53 indicates chronic inflammation

We investigated whether cancers carrying mtp53 are more inflamed than their wtp53 counterparts. We identified differentially expressed genes (DEGs) in breast cancer with mtp53 relative to wtp53 by using RNA-Seq data deposited in TCGA. Next, we performed gene set enrichment analysis (GSEA) on the upregulated genes (log2FoldChange > 1, *p*adj > 0.05) from DEGs. We found that the gene sets involved in virus infection and cytokine signalling (Fig. S1a, marked in blue), gene sets associated with autoimmune disease (Fig. S1a, green), as well as gene sets related to innate and adaptive immune responses (Fig. S1a, orange) were significantly upregulated in mtp53 BRCA compared to wtp53 BRCA, indicating ongoing inflammation status within TME of breast cancers with mtp53. These results are partially in line with the previous findings [28, 29]. We would like to note, that the gene expression patterns could be from tumour cells, immune cells and stromal cells, which is difficult to differentiate using RNA-seq analysis.

**Fig. 1:**
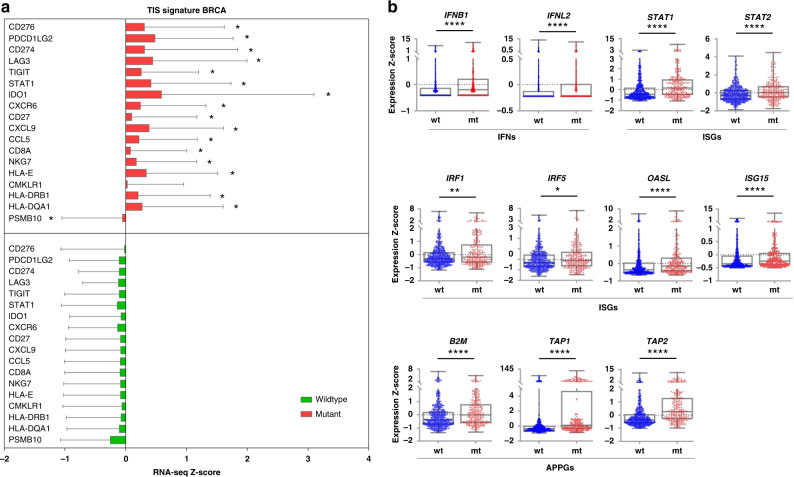
Elevated TIS signature and IFN signalling in breast cancer patients (TCGA-BRCA) carrying mtp53. **a** Expression of a majority of TIS signature genes was significantly upregulated in breast cancer patients carrying mtp53. Bar graphs were generated by graphpad prism: the expression of z-score of each gene in the TIS signature genes in either mtp53 or wtp53 TCGA-BRCA. Error bars represent the standard deviation of expression of a gene across breast cancer patient samples. **b** Interferons (IFNs), interferon-sensitive genes (ISGs) and antigen processing and presentation genes were upregulated in breast cancer patients carrying mt p53, as shown in the box and whiskers plot (whiskers from min to max, shown are all data points). **a**, **b** Patient samples carrying p53 missense mutation or in frame deletion/insertion were grouped into p53 mutant. There were 618 patients carrying wtp53 and 258 patients carrying mtp53. *p* value is two-tailed and centre values are median. Mann–Whitney test. *p ≤ 0.05, **p ≤ 0.01, ***p ≤ 0.001, ****p ≤ 0.0001.

To further corroborate the GSEA results, we evaluated the TIS score, the 18-gene signature that determines the pre-existing but suppressed adaptive immune response within tumours [30]. We analysed the expression of genes associated with IFN signalling, a major inflammatory pathway that bridges the innate and adaptive immune responses [9], using RNA-Seq data from breast cancer patients in TCGA. Our analysis revealed a significantly higher expression of the major TIS genes in breast cancer with mtp53 relative to wtp53 (Fig. 1a), suggesting that breast cancers which have lost wtp53 function are more inflamed. This finding also implies that these tumours have developed mechanisms to escape from immune surveillance.

Next we analysed the expression of inflammation-related genes in a more detail. We found an enhanced IFN signalling in breast cancer patients with mtp53, including an increased expression of *IFNλ2*, type III IFN, and *IFNβ*, type I IFN, as well as interferon-sensitive genes (*ISG*s) downstream of IFN (Fig. 1b). These include *STAT1*, *STAT2*, *IRF1* and *IRF5*, which form the transcriptional complex and promote transcription of genes involved in the IFN response [31], and *OASL* and *ISG15*, which can further boost the IFN response [32, 33]. We also found an increased expression of antigen processing and presentation genes downstream of the IFN signalling, including *B2M*, *TAP1* and *TAP2* in mtp53 BRCA (Fig. 1b). The products of these genes facilitate the presentation of tumour-derived neo-antigens on the surface of tumour cells, potentially promoting more efficient recognition and killing by cytotoxic T cells.

In summary, our results suggest that the loss of wtp53 function is linked to the presence of chronic inflammation within BRCA patients, which may contribute to cancer progression.

### Mutant p53 mediates the elevated expression of the IFN pathway and endogenous retroviruses (ERVs) in cancer cell lines

Elevated IFN response, in breast cancer patients with mtp53 could be due to the loss of function (LOF) of wtp53 and/or GOF of mtp53. We addressed the question, whether mtp53 GOF contributes to the increased IFN signalling. We assessed the expression of IFN pathway by RT-qPCR upon shRNA-mediated depletion of mtp53 in eight human and one mouse cancer cell lines of different origin. We used lentivirus containing p53 shRNA along with GFP gene to detect transduced cells and confirmed efficient transduction by immunofluorescence and p53 knockdown by western blot (Fig. S2a, b).

**Fig. 2:**
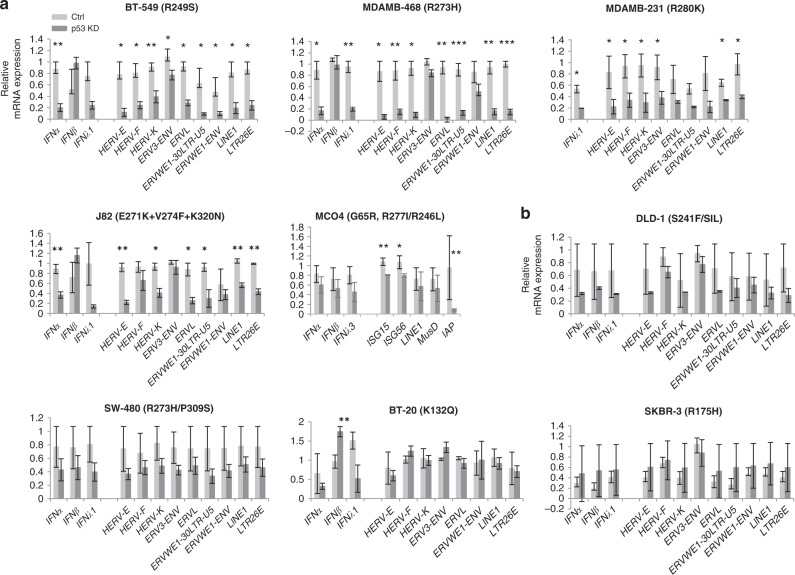
Mtp53-dependent elevated IFN signalling and ERVs expression in cancer cell lines. **a** Downregulation of type I and/or type III IFNs, and ERVs after knockdown of mtp53 in p53 mutant cancer cell lines, as assessed by qPCR (*n* = 3). Human breast cancer cell lines: BT-549, MDAMB-468 and MDAMB-231; human bladder cancer cell line, J82 and mouse fibrosarcoma MCO4. **b** Knockdown of mtp53 did not affect expression of IFNs and ERVs in another set of p53 mutant cancer cell lines, as assessed by qPCR (*n* = 3). Human colorectal cancer cell lines: DLD-1 and SW-480; human breast cancer cell lines: BT-20 and SKBR-3. **a**, **b** Each condition contains three biological repeats and each biological repeat includes three technical repeats. Unpaired student t-test, Centre values are mean, Error bars, s.d. *p* value is two-tailed. *p ≤ 0.05, **p ≤ 0.01, ***p ≤ 0.001.

We found that the expression of IFN genes, either *IFNα* or *IFNλ1*, was significantly reduced upon mtp53 depletion in breast cancer cell lines BT-549, MDAMB-468, MDAMB-231, a bladder cancer cell line J82 and mouse fibrosarcoma MCO4 (Fig. 2a). These data support the notion that mtp53 can mediate the induction of the IFN pathway.

Previous studies, including ours, have provided substantial evidence that the induction of expression of endogenous retroviruses (ERVs), followed by accumulation of dsRNA, could be an underlying cause of activation of IFN signalling via viral mimicry signalling [23, 34–36]. Therefore, to investigate the possible mechanisms of IFN pathway induction by mtp53, we analysed whether the expression of ERVs in these cell lines is dependent on mutant p53. Notably, we found a substantial reduction of ERVs and LINE1 expression upon p53 depletion (Fig. 2a). These results suggest that in contrast to wtp53 loss of function, which has been shown to induce a partial de-repression of ERVs [37], some p53 mutants can enhance the expression of ERVs, presumably leading to the induction of IFN pathway. This is in accordance with the recent finding that ovarian cancer cells with mtp53 had enhanced basal level expression of repetitive elements including ERVs [38]. Hence, mutant p53-dependent elevated levels of ERVs and LINE1 expression might reflect a novel GOF of mtp53.

However, we did not observe a significant decrease of IFNs and ERVs, as well as LINE1 expression upon p53 depletion in some cell lines, including breast cancer cell lines BT-20, SKBR-3 and colon cancer cell lines DLD-1 and SW480 (Fig. 2b). These data suggest that this novel mtp53 GOF might be context-dependent, which is in line with previous studies on mtp53 GOF [39].

### Enhanced cytotoxic T lymphocyte (CTL) infiltration in breast cancers expressing mtp53

**Fig. 3:**
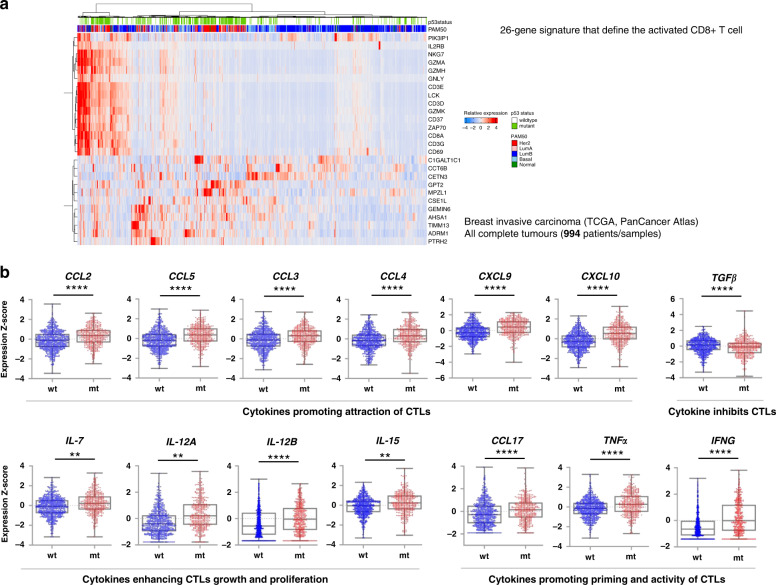
Analysis of gene expression patterns reveals enhanced CTLs infiltration in breast cancers (TCGA-BRCA) carrying mtp53. **a** Hierarchical clustering showed that mtp53 patient samples displayed higher expression (mRNA z-score) of 26-gene signature that defines activated CD8+ T cells cluster together. **b** In comparison to patients carrying wtp53, tumour samples from patients carrying mtp53 had elevated expression of cytokines that promote attraction, priming and activity of CTLs, and cytokines that enhance growth and proliferation of CTLs as well as decreased expression of TGFβ, a cytokine that inhibits CTL function, as shown in the box and whiskers plot (whiskers from min to max, shown are all data points). **a**, **b** Patient samples carrying p53 missense mutation or in frame deletion/insertion were grouped into p53 mutant. There were 618 patients carrying wtp53 and 258 patients carrying mtp53. *p* value is two-tailed and centre values are median. Mann–Whitney test. **p ≤ 0.01, ****p ≤ 0.0001.

Having found elevated TIS score in mtp53 breast cancers, we decided to check the abundance and functional properties of CD8+ T cells within tumour. We compared the expression of CD8+ T cells-related markers in mtp53 and wtp53 breast cancers, since CD8+ T cells are major effector of the adaptive immune response. We detected a tendency for a higher expression of 26-gene signature that define the activated CD8+ T cell [40] in mtp53 breast cancers compared to wtp53 ones, as can be seen in the heatmap (Fig. 3a). Twenty genes out of these 26 signature genes were significantly upregulated in breast cancers carrying mtp53 (Fig. S3a). These include several markers involved in TCR signalling propagation, such as *CD8A*, *LCK*, *CD3D*, *CD3E*, *CD3G* and *ZAP70* and also a number of markers implicated in cytotoxicity of CD8+ T cells, like *GZMH*, *GZMK*, *NKG7*, *GNLY* and *IL2RB*.

We observed a significant upregulation of a gene set “cytokine-cytokine receptor interaction” in mtp53 breast cancers (Fig. S1a) and reasoned that this could be the underlying cause for the enhanced infiltration of activated CD8+ T cells. Therefore, we analysed the expression of cytokines related to CD8+ T cell function. We found that the expression of a number of cytokines was enriched in breast cancers with mtp53 (Fig. 3b). These include cytokines which facilitate the infiltration of CD8+ T cells, *CCL2*, *CCL3*, *CCL4*, *CCL5*, *CXCL9* and *CXCL10*; cytokines engaged in the growth and proliferation of CD8+ T cells, *IL-7*, *IL-12* and *IL-15*; cytokines involved in the priming and activity of CD8+ T cells, *IL-17* and *TNF-α*, and *IFN-γ*, respectively, while *TGF-β*, the inhibitory cytokine of CD8+ T cells was decreased in mtp53 breast cancers (Fig. 3b).

Taken together, our data suggest that both the abundance and functional polarisation of CD8+ T cells were enhanced in mtp53 breast cancers in contrast to wtp53 breast cancers. The presence of infiltrated active CD8+ T cells in developed tumours suggests that mtp53 tumour cells evolved mechanisms allowing them to escape from immune surveillance.

### Mechanisms of escape from immune surveillance in mutant p53 tumours

**Fig. 4:**
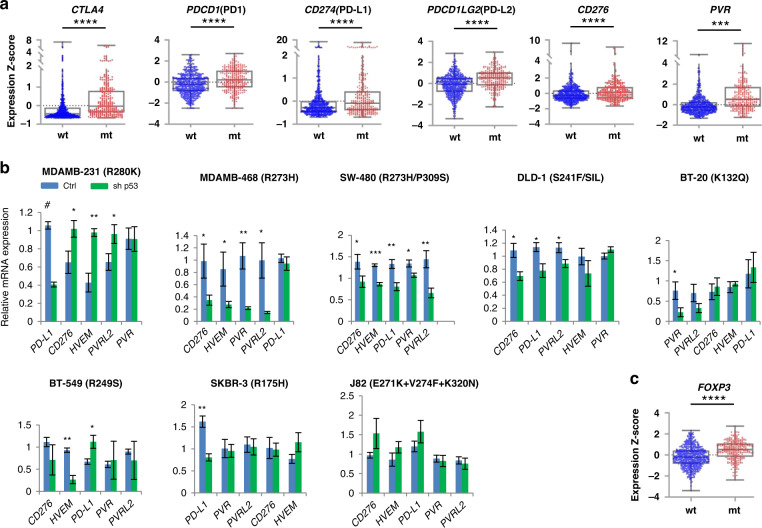
Upregulation of immune checkpoints as possible mechanism of mtp53 breast cancers escape from immune surveillance. **a**, **c** Breast cancer samples from patients (TCGA-BRCA) carrying mtp53 had increased expression of immune checkpoint molecules (**a**), including CTLA4, PD1, PD-L1, PD-L2, CD276 and PVR, and FOXP3 (**c**), a well-established Treg cell marker. Figures were shown as the box and whiskers plot (whiskers from min to max, shown are all data points). **a**, **c** Patient samples carrying p53 missense mutation or in frame deletion/insertion were grouped into p53 mutant. There were 618 patients carrying wtp53 and 258 patients carrying mt p53. *p* value is two-tailed and centre values are median. Mann–Whitney test *p ≤ 0.05, **p ≤ 0.01, ***p ≤ 0.001, ****p ≤ 0.0001. **b** Immune checkpoint molecule expression before and after p53 knockdown in cancer cell lines. Human breast cancer cell lines: MDAMB-231, MDAMB-468, BT-20, BT-549 and SKBR-3; human colorectal cancer cell lines: SW-480 and DLD-1; human bladder cancer cell line: J82. Each condition contains three biological repeats and each biological repeat includes three technical repeats. Unpaired Student’s *t* test, centre values are mean, Error bars, s.d. *p* value is two-tailed. *p ≤ 0.05, **p ≤ 0.01, ***p ≤ 0.001, ^#^*p* ≤ 0.0001.

Next we set up to explore, what is the mechanism(s) of immune surveillance escape in mtp53 breast cancers with enhanced TIS score (Fig. 1a) and increased CD8+ T cells infiltration (Fig. 3a, b). We hypothesised that both tumour intrinsic and extrinsic mechanisms could contribute. The expression of immune checkpoint molecules on the surface of tumour cells is an important strategy for tumour cells to escape from CD8+ T cell-mediated surveillance [41]. We thus analysed the expression of several well studied inhibitory molecules and found that *CTLA-4*, *PD-1*, *PD-L1*, *PD-L2*, *CD276* and *PVR* were upregulated in breast cancer patients with mtp53 (Fig. 4a). This is consistent with previous findings that acute myeloid leukaemia (AML), and breast cancers in METABRIC database carrying mtp53 had higher expression of immune checkpoints than their wtp53 counterparts [28, 42]. It is possible, that the high level of immune checkpoint molecules contribute to the mtp53 tumour cell survival in spite of increased infiltration of activated CD8+ T cells.

Taking into account our analysis of patient data, we decided to test whether the upregulation of immune checkpoints might be mediated by mtp53. We found that mtp53 depletion resulted in downregulation of several immune checkpoints, including PD-L1 (*CD274*), B7-H3 (*CD276*), PVR (*CD155*), PVRL2 (*CD112*), and *HVEM* (Fig. 4b). Depending on the cell line, depletion of mtp53 leads to downregulation of different checkpoints, numbering from one to five. These data support our results obtained by analysis of patient samples and suggest that mtp53 is involved in the upregulation of immune checkpoint molecules (Fig. 4b).

T regulatory (Treg) cells play important roles to suppress immune response, including CD8+ cytotoxic T lymphocyte activity [43]. We examined whether the expression of *Foxp3*, a well-established Treg cell marker, was elevated in mtp53 tumours. We found that *Foxp3* was expressed significantly higher in breast cancers with mtp53 than in wtp53 breast cancers (Fig. 4c), in accordance with a similar finding in AML [42]. This indicates an enhanced Treg cell infiltration within the TME of mtp53 breast cancer patients, with capacity to suppress the activity of CD8+ T cells.

In summary, we find that breast cancers with mtp53 might escape from elevated immune surveillance via upregulation of negative immune checkpoints and infiltration of Treg cells within TME.

### APR-246 represses immune checkpoints and activates IFN signalling in p53 mutant cancer cell lines

**Fig. 5:**
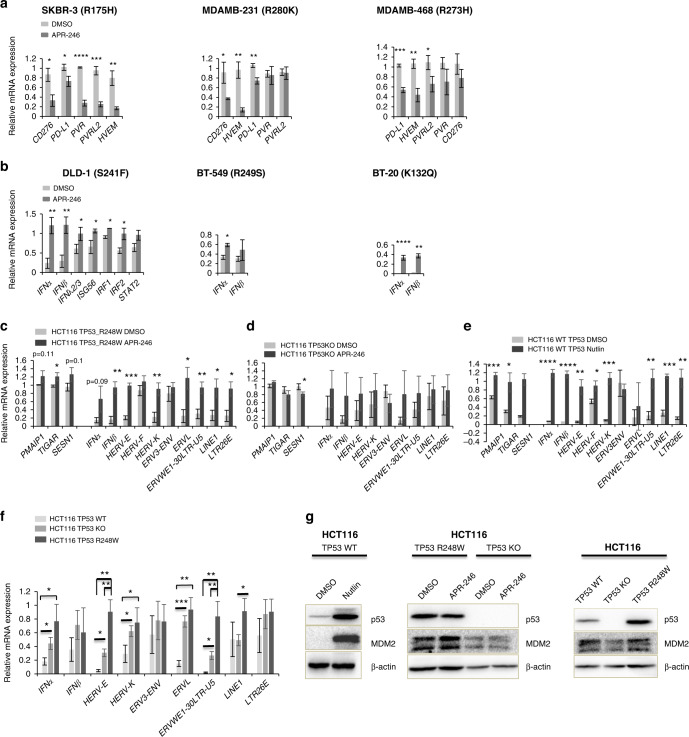
APR-246 repressed the expression of immune checkpoints and activated IFN signalling in p53 mutant cancer cells. **a** APR-246 repressed the expression of immune checkpoint molecules in cancer cell lines, as assessed by qPCR (*n* = 3). Human breast cancer cell lines: SKBR-3, MDAMB-231 and MDAMB-468. **b** APR-246 enhanced the expression of IFN signalling genes in cancer cell lines, as assessed by qPCR (*n* = 3). Human colorectal cancer cell line: DLD-1; human breast cancer cell lines: BT-549 and BT-20. **c** Induction by APR-246 of ERVs and IFN genes along with p53 target genes *PMAIP1*, *TIGAR* and *SESN1* in HCT116 cells expressing p53 mutant R248W, as assessed by RT-qPCR (*n* = 3). **d** ERVs and IFN genes and p53 target genes *PMAIP1*, *TIGAR* and *SESN1* are not significantly affected by APR-246 in p53-null HCT116 cells. **e** Induction by MDM2 inhibitor nutlin of ERVs and IFN genes along with p53 target genes *PMAIP1*, *TIGAR* and *SESN1* in HCT116 cells expressing wtp53, as assessed by RT-qPCR (*n* = 3). **f** Comparison of basal levels of ERVs and IFN genes in isogenic HCT116 cells with different p53 status: wt, mutant and null, as assessed by RT-qPCR (*n* = 3). **g** Western blot assessment of the levels of p53 and MDM2 in wtp53 HCT116 cells treated/non-treated with nutlin (left panel), in HCT116p53R248W and HCT116p53null cells treated with APR-246 (middle panel) and in non-treated wtp53HCT116, p53null HCT116 and p53 mutant R248W-expressing HCT116 cells (right panel). **a**–**e** Each condition contains three biological repeats and each biological repeat includes three technical repeats. Unpaired Student’s *t* test, centre values are mean, error bars, s.d. *p* value is two-tailed. *p ≤ 0.05, **p ≤ 0.01, ***p ≤ 0.001, ****p ≤ 0.0001. Treatment conditions were as follows: 12 h treatment of 40μM APR-246 on SKBR-3 and BT-549, 12 h treatment of 200μM APR-246 on MDAMB-231, 12 h treatment of 60 μM APR-246 on MDAMB-468, 24 h treatment of 90 μM APR-246 on DLD-1, 12 h treatment of 100 μM APR-246 on BT-20, 24 h treatment of 20 μM APR-246 on both HCT116p53R248W and HCT116p53null cells, 24 h treatment of 10 μM nutlin on wtp53HCT116 cells.

Targeting immune checkpoints by anti-PD1/PD-L1 and anti-CTLA4 antibodies has revolutionised anti-cancer therapy [44]. Monoclonal antibodies targeting several other checkpoint molecules are emerging and demonstrate good results in preclinical models and in clinical trials [19, 20, 22]. Taking into account our finding that one of the possible ways of mtp53 cancers to escape from immune control is upregulation of immune checkpoints, we decided to assess whether the mutant p53 restoration can reverse the upregulation of immune checkpoints. We tested whether mtp53 reactivating compound APR-246 can regulate the expression of inhibitory immune checkpoints, involving PD-L1 (*CD274*), B7-H3 (*CD276*), PVR (*CD155*), PVRL2 (*CD112*), and *HVEM*. We found that the expression of at least three different checkpoint molecules was suppressed upon APR-246 treatment in breast cancer cell lines SKBR-3, MDAMB-231 and MDAMB-468 (Fig. 5a), while at least one checkpoint molecule was repressed in four other cancer cell lines (Fig. S4a). Thus, the inhibition of immune checkpoints by APR-246 might overcome tumour intrinsic inhibitory mechanisms of T cell-mediated tumour cell killing.

IFNs can suppress tumours via direct induction of apoptosis or promotion of anti-tumour immunity [45–47]. Thus, we reasoned that in cancer cells with low IFN expression the anti-tumour effect could be enhanced by induction of IFN pathway. As shown in Fig. 5b, APR-246 treatment induced type I IFN (*IFNα* & *IFNβ*), type III IFN (*IFNλ2/3*) and some downstream interferon sensitive genes (ISGs) in human colorectal cancer cell line DLD-1 and human breast cancer lines BT-20 and BT-549.

Of note, APR-246-mediated upregulation of IFNα and the downstream target STAT2 was mtp53-dependent, as well as the induction of p53 target genes p21 (*CDKN1A*) and Noxa (*PMAIP1*) as shown in the DLD-1 cancer cell line (Fig. S4c). In addition, the p53 target gene *PMAIP1* was activated by APR-246 in seven different cancer cell lines expressing mtp53 (Fig. S4d). Moreover, the p53 target MDM2 was induced by APR-246 on protein level in a mtp53-dependent manner in H1299p53R175H cells (Fig. S4h) and in HCT116p53R248Q cells (Fig. 5g). APR-246-mediated induction of p53 target genes *PMAIP1* (Noxa), *TIGAR* and *SESN1* was observed only in the presence of mtp53-R248W expression (Fig. 5c).

To provide more clues as to the mutp53 GOF in induction of IFN pathway, we performed a series of experiments to assess the expression of ERVs in isogenic cell lines with different p53 status: expressing mutant p53 (Tet-regulatable R175H and stably transfected R248W), p53-null and wtp53. We used two different cell systems, based on p53-null H1299 lung adenocarcinoma and colon carcinoma HCT116 expressing wtp53, and two compounds that reactivate p53. APR-246 that restores mutant p53 and the MDM2 inhibitor nutlin that activates wtp53. Our results from the analysis of the HCT116 cell system (Fig. 5c–g) and for the H1299 cell system (Fig. 4e–h), confirmed the induction of ERVs and IFN genes as GOF by p53-R175H and p53-R248W mutants. Restoration of wt function for mutant p53 by APR-246 (as evidenced by the induction of p53 target genes mentioned above) resulted in further induction of ERVs and IFN genes (Fig. 5c and Fig. S4f). This is in line with the induction of ERVs and IFN genes upon wtp53 activation by nutlin (Fig. 5e), as we have found previously [23]. The comparison of ERVs and IFN genes in isogenic HCT116 wtp53, HCT116 p53-null and HCT116 p53-R248W cell lines demonstrated that the deletion of p53 led to a partial de-repression of ERVs and IFN genes, in line with previous findings that wtp53 represses ERVs at basal conditions [37]. Mtp53 R248W expression in HCT116p53-null cells led to a further de-repression of ERVs and IFN genes, corroborating its GOF activity (Fig. 5f).

Taken together, our data suggest that restoration of wt function for mtp53 by APR-246 boosted the IFN response in various types of cancer cell lines.

It is worth noting that APR-246 did not induce IFN signalling in several other cancer cell lines including breast carcinoma MDAMB-231, MDAMB-468 and SKBR-3 cells (Fig. S4b). On the other hand, APR-246 repressed the expression of three to five different immune checkpoints in these cell lines (Fig. 5a). In the majority of cell lines in which APR-246 induced the IFN response, we did not observe the repression of immune checkpoints. However, in those cell lines in which APR-246 treatment did not induce IFN expression, we detected a substantial repression of immune checkpoints. These data suggest that tumour cells with mtp53 can evolve alternative mechanisms of escape from immune surveillance: upregulation of immune checkpoints in tumours with high IFN signalling or downregulation of IFN signalling.

In summary, we find that APR-246 disables the mtp53-mediated tumour cells evasion from immune surveillance either via upregulation of the IFN pathway or via downregulation of inhibitory immune checkpoints, depending on the mechanisms of immune escape in a particular type of cancer cells.

### APR-246 can promote CD4+ T cells infiltration and decrease the exhaustion of CD8+ T cells in vivo

**Fig. 6:**
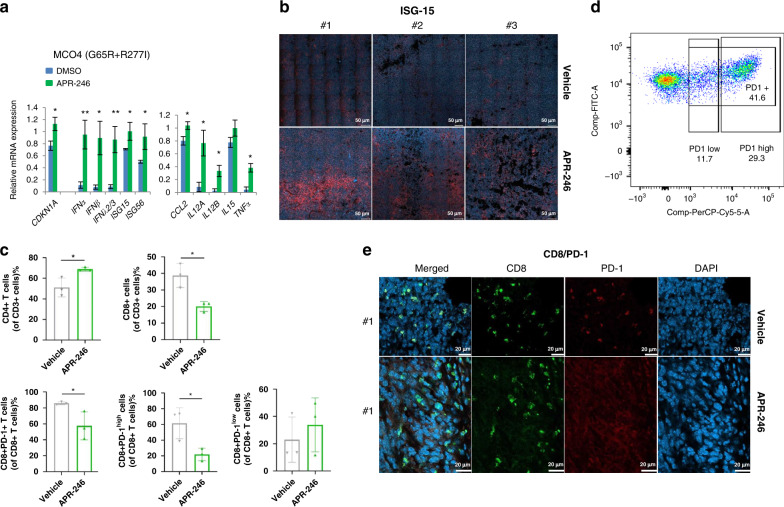
APR-246 promoted CD4+ T cell infiltration and decreased the exhaustion of CD8+ T cells in a p53 mutant MCO4 fibrosarcoma syngeneic mouse model. **a** APR-246 activated the expression of IFN signalling genes and cytokines that enhance the production and migration of T cells in a murine fibrosarcoma cancer cell line MCO4. Each condition contains three biological repeats and each biological repeat includes three technical repeats. Unpaired Student’s *t* test, centre values are mean, error bars, s.d. *p* value is two-tailed. *p ≤ 0.05, **p ≤ 0.01. Treatment conditions: 24 h treatment of MCO4 cells with 10 μM APR-246. **b**, **e** IHC detection of ISG-15, CD8 and PD-1 on tumour samples after either Vehicle or APR-246 treatment. **c** Flow cytometry detection of CD4+ T cells, CD8+ T cells, CD8+ PD-1+ T cells, CD8+ PD1^high^ T cells and CD8+ PD1^low^ T cells within tumour microenvironment after either Vehicle or APR-246 treatment. **d** Gating strategy of CD8+ PD1^high^ and CD8+ PD1^low^ T cells.

Our results showed that APR-246 induced IFNs in different types of cancer cell lines (Fig. 5b), including murine fibrosarcoma MCO4 (Fig. 6a), which express two mtp53 species, one with G65R and R277I mutations and the other has R246L mutation [48]. Moreover, APR-246 upregulated cytokines that enhance T cell production and migration, including *CCL2, IL12* isoform A and B and *TNFα* (Fig. 6a). Thus, we examined whether APR-246 could promote immune cells infiltration and function in a MCO4 syngeneic Balb/c mouse model.

When tumour volume reached around 300 mm^3^, we applied APR-246 by i.p. injection with a dosage of 100 mg/kg [25], and performed FACS analysis of immune cell populations. The treatment schedule for both experiments is shown in Fig. S5a. The antibody panels, immune cell populations identified and corresponding identification markers are listed in Supplementary Table S1. The FACS gating strategy for immune cell populations is shown in Fig. S5b.

The IFN pathway was induced by APR-246 in vivo, as evidenced by the increased ISG-15 staining in tumours (Fig. 6b). We observed a significantly higher ratio of CD4+ T cell infiltration in APR-246-treated mice than in vehicle-treated mice (Fig. 6c, upper panels). CD4+ T cells enhance the immune response in many ways, including facilitation of CD8+ T cells activation and proliferation, boosting the production of antibodies by B cells and direct anti-tumour cytotoxicity [49]. Thus, our data indicate that APR-246 stimulated a CD4+ T cell mediated anti-tumour immune response.

Next we examined the CD8+ T cell populations and observed a lower ratio of these cells in APR-246 treated mice than in control mice (Fig. 6b, lower panels). However, over 80% of CD8+ T cells were PD-1 positive in the control group as compared to lower than 60% in APR-246 treated mice.

We further classified CD8+ T cells into PD-1 high or PD-1 low populations (Fig. 6d). In the control group, 60% of all CD8+ T cells expressed high level of PD-1, whereas only 20% of all CD8+ T cells were PD-1 high in APR-246-treated mice. There was no significant difference of PD-1 low populations between the two treatment groups (Fig. 6b). These data were corroborated by lower level of PD-1-positive CD8+ T cells in tumours treated with APR-246, as demonstrated by immunohistochemical staining of CD8+ cells with anti-PD-1 antibody (Fig. 6e and Fig. S5e, f).

Taken together, our results strongly suggest that APR-246 prevents the exhaustion of CD8+ T cells within the TME.

As a control for APR-246 activity, we observed a significant tumour suppression and induction of apoptosis by APR-246 in the absence of weight loss of animals (Fig. S5c, d), in accordance with the previous study [25].

In summary, we found that APR-246 induced IFN signalling, facilitates the infiltration of CD4+ T cells and counteracts the exhaustion of CD8+ T cells in the MCO4 mouse model.

## Discussion

Our analysis of RNA-seq data from breast cancer patients deposited in TCGA revealed that breast cancers with mtp53 have a propensity to enhanced inflammation, exemplified by elevated IFN signalling, TIS score and infiltration of CD8+ T cells. We identified a novel GOF of mtp53—it’s ability to upregulate ERVs, IFNs and immune checkpoints in a context-dependent manner. We found that APR-246 could disable mtp53-mediated mechanism of cancer cell escape from immune surveillance via activation of IFN signalling or repression of negative immune checkpoint molecules in mtp53 cancer cell lines. Moreover, APR-246 promoted CD4+ T cell infiltration and prevented CD8+ T cell exhaustion in the MCO4 syngeneic mouse model.

It is well established that chronic inflammation can lead to cancer initiation and progression by creating a favourable TME [1]. In recent years, the ability of mtp53 to promote inflammation via GOF has attracted considerable attention. There are a number of ways by which mtp53 can contribute to inflammation. Cancer cells expressing GOF p53 mutants, including R175H, H179I, R273H and D281G, upregulate inflammatory CXC chemokines [50], while some p53 mutants can boost TLR3-mediated cytokine expression [51]. Moreover, mtp53 has been shown to facilitate the inflammatory response via repression of the anti-inflammatory cytokine, interleukin-1 receptor antagonist (IL1RN) [52]. Furthermore, mutant p53 interacts directly with NF-κB, the major player in inflammation [5]. Interaction of mtp53 with NF-κB subunits p50 and p65 increases their nuclear translocation or prolongs the transcriptional activity of NF-κB, which helps to promote and maintain chronic inflammation status [52, 53]. However, there is more to learn about pro-inflammatory function of mtp53.

Our study reveals a novel mtp53 GOF activity, its ability to mediate elevated levels of IFNs (Fig. 2a), contributing to the enhanced inflammation signalling in mtp53 breast cancers. While our results presented in Figs. 5 and S4 firmly established the induction of ERVs and IFN genes as a novel mutant p53 GOF, it appears paradoxical that both wtp53 activated by MDM2 inhibitor and mutant p53 induced the expression of ERVs and IFN genes. Moreover, their expression is further increased by reactivation of mutant p53 by APR-246.

In our recent paper [23] we show that wtp53 activated by MDM2 inhibitors induces the expression of ERVs, in contrast to the basal function of p53, which is to repress them. We have suggested a model, implying that at basal condition wtp53 is present on ERVs promoters together with corepressors DNMT1 and LSD1. Upon p53 activation high levels of p53 increase its occupancy on ERVs promoters simultaneously with sequestration and downregulation of DNMT1 and LSD1, which allows p53 to activate the expression of ERVs.

To explain our seemingly contradictory finding that mtp53 de-represses ERVs, we speculate that, since mutant p53 can not bind DNA but can interact with the same transcription factors as wtp53 and due to its high level in cells can sequester corepressors of ERVs. This will lead to ERVs de-repression, to a higher extent than simply wtp53 deletion. Upon restoration of wt function to mutant p53 by APR-246, restored p53 actively induces ERVs by binding to their promoters. This idea is illustrated in our model in Fig. S6.

Interestingly, a recent study has demonstrated that p53 GOF phenotypes are independent on p53 alteration, but depend on the cell context [54]. It is indeed the case in our study: the same hot spot mutant R273H displayed GOF in MDA-MB468 cells, but not in SW480 cells (Fig. 2a, b).

Recent study demonstrates that mtp53 R249S suppresses IFNB1 production in the BT549 triple negative breast cancer (TNBC) cell line via inhibition of TBK1-STING-IRF3 pathway [10]. In line with this study, we observed a tendency of IFNB1 increase upon mtp53 depletion. However, IFNA1 was significantly downregulated in several breast cancer cell lines after mtp53 depletion, with 4-fold decrease in BT549 cells (Fig. 2a). These data indicate that mtp53 might regulate IFNA1 and IFNB1 via different mechanisms. In addition, some correlation (*p* = 0.042) of IFNB1 mRNA with wtp53 rather than mtp53 was found in triple negative breast cancer samples [10]. In contrast, we found a significantly higher expression of IFNB1 and IFNL2 as well as downstream signalling in mtp53 cancers as compared to wtp53 cancers by analysing all human breast cancer RNA-Seq data in TCGA. We believe that the difference with the previous study [10] might be context-dependent, affected by cancer subtypes and stages.

Type I and/or type III IFNs and their upstream stimulators, ERVs, were downregulated after mtp53 knockdown in several cancer cell lines. The exact mechanism of this GOF by mtp53 is not clear yet, while the context dependence of it suggests the involvement and availability of some unknown cofactors. There are a number of candidate TFs and microRNAs which could be involved in changes of expression of immune genes that we have found to be deregulated in a mutant p53-dependent manner. TFs bound by GOF p53 mutants include, among others, p63, p73, NF-kb, STAT3, SREBPs, SP1, ETS1/2, E2F1/4, NRF2, c-Myc, NF-Y, VDR, FOXO1 [55]. A search for TFs that are hijacked by mutant p53 has culminated recently in discovery of hnRNPK and CREB1 as mediators of mutant p53 GOF [56, 57]. To explain the elevated level of inflammatory genes in mtp53 cancers, we favour the hypothesis described above, implying that mutant p53 sequesters repressors of ERVs, thereby leading to their de-repression, which in turn triggers IFN genes and inflammatory signalling. Future studies will help to identify the exact molecular mechanisms involved in this novel mutant p53 GOF.

Chronic IFN activation status within cancer cells has been linked to the production of a proinflammatory and tumour-promoting microenvironment [58], as well as resistance to immune checkpoint therapy [59]. High throughput study of the immune landscape of cancers [29] using the RNA-seq data from TCGA for 33 cancer types identified *TP53* mutated cancers of diverse cancer types to be associated with increased lymphocyte fraction. These include breast carcinoma, head and neck carcinoma, gastric and ovarian carcinoma. Further, *TP53* mutations have been found to correlate with increased immune infiltration and to associate with remarkable clinical benefit to anti-PD-1 therapy in lung adenocarcinoma and acute myeloid leukaemia [42]. A recent study on *TP53* hotspot mutations in acute myeloid leukaemia showed that cancers with mutant *TP53* exhibited higher interferon signalling, more infiltrating immune cells, and stronger response to immunotherapy [42].

Recent findings have established that epigenetic drugs and targeted compounds that lead to acute IFN activation showed impressive tumour suppression, infiltration of anti-tumour immune cells [23, 35, 36], as well as increased sensitivity to immune checkpoint blockade [35, 60, 61]. Therefore, we reasoned that the tumour immune surveillance could be recovered if we can further boost the IFN pathway in the mtp53 tumour cells. Our data suggests that restoration of mtp53 function by APR-246 might induce acute IFN activation, which can boost infiltration of CD8+ T cells and negate the expression of immune checkpoints.

We discovered that APR-246 could induce type I and/or type III IFN expression in a number of mtp53 cancer cell lines (Fig. 5b) and repress negative immune checkpoint molecules in a number of mtp53 cancer cell lines (Figs. 5a and S4a), which potentially increases the sensitivity of tumour cells to CD8+ T cells. Both type I and III IFN are known to induce tumour apoptosis, enhance antigen processing and presentation in tumours, facilitate the production of pro-inflammatory cytokines in DCs and inhibition of Tregs [9, 62, 63], thus promoting immune cell-mediated killing of tumour cells.

Several immune checkpoints or T cell inhibitory receptor ligands (TCIRLs) were repressed upon APR-246 treatment of mtp53 cancer cell lines (Fig. 5a and Fig. S4a). Downregulation of TCIRLs might be due to IFN repression by APR-246 in these cell lines (Fig. S4b) [63]. It is interesting to note that after APR-246 treatment, cell lines that responded by induction of IFNs, do not have impressive inhibition of immune checkpoints and vice versa, implying that APR-246 can boost immune response via different mechanisms depending on the cellular context. Since co-expression of multiple TCIRLs is related to resistance to immune checkpoint therapy [59], APR-246 might be a good candidate for combination therapy with immune checkpoint blockade. The combination of immune checkpoint inhibitor permbrolizumab with APR-246 is currently being tested in a clinical trial in solid tumours (ClinicalTrials.gov identifier NCT04383938).

We evaluated the idea that APR-246 can affect immune response within the TME by using the MCO4 fibrosarcoma syngeneic mouse model, carrying two different mtp53 species [25]. Previous studies have demonstrated that APR-246 can suppress tumour growth in several preclinical in vivo models [25, 64], and we also confirmed the tumour suppression effect of APR-246 in our study (Fig. S5c). Furthermore, we found significantly higher number of CD4+ T cells, but, to our surprise, lower number CD8+ T cells in APR-246-treated tumours. We observed that in control tumours, 60% of CD8+ T cells were exhausted, as indicated by higher PD-1 expression, in contrast to only 20% of T cells in tumours isolated from APR-246 treated mice. We discovered that APR-246 increased the total number of CD4+ helper T cells within the TME, making it possible to boost the function of other immune cells [49].

Interestingly, in a recent report at American Association for Cancer Research (AACR) Annual Meeting, Atlanta, 2019, Arnab and co-workers using wtp53 B16 model, showed that APR-246 can promote infiltration of M1 polarised macrophages and Foxp3+ Treg cells, increase Granzyme B activity of CD8+ T cells and upregulate PD-L1 expression in macrophages, and PD-1 expression in CD8+ T cells within the TME. Moreover, the combination of APR-246 with anti-PD-1 antibody significantly suppressed the tumour growth and prolonged survival in comparison to monotherapies in both B16 melanoma and mtp53 MC38 colorectal tumour-bearing mouse models. The authors confirmed that this anti-tumour effect of combination therapy was abolished in mice deficient in T cells and therefore was T cell-dependent. In fact, the analysis of the TME revealed that this combination therapy reduced proliferation but enhanced functional activity of CD8+ T cells [64].

The exact mechanisms how APR-246 affects the immune cell infiltration and their functional polarisation within the tumour remain to be identified. In order to gain more insight, it would be important to assess, whether and how APR-246 alters cytokine profiles within TME, immune cell populations within spleen and metabolic reprogramming of immune cells, as well as to learn more how APR-246 affect the functional properties of CD8+ T cells and if functional markers, like IFNγ, Granzyme B and Perforin are involved.

IFNs are essential for the induction of anti-cancer immune response and infiltration as well as functional sustainability of lymphocytes within TME, and are critical for successful tumour immune surveillance. APR-246 is now under extensive testing in clinical trials (ClinicalTrials.gov) on both liquid and solid cancers in combination with several chemotherapeutic and targeted agents. Our results uncovered the favourable effects of APR-246 on anti-cancer immune surveillance, suggesting that the combination of APR-246 with immune therapy could be beneficial for patients.

## Supplementary information

Supplementary Figures

Supplementary Method

Antibodies panels and immune cells identification markers for immunoprofiling of tumour infiltrating immune cells

qPCR primers and shRNA sequence targeting human p53

Reproducibility checklist

## Data Availability

The data that support the findings of this study are available from cBioPortal for Cancer Genomics and from TCGA-BRCA project of GDC Data Portal.
