# Supplementary material for: Mutant p53 gain of function mediates cancer immune escape that is counteracted by APR-246
Source: Br J Cancer. 2022 Sep 22;127(11):2060–71. doi: 10.1038/s41416-022-01971-8 (PMC9681866; doi:10.1038/s41416-022-01971-8)
Supplement: Supplementary file 1 — Supplementary Figures [file 41416_2022_1971_MOESM1_ESM.pptx]

## Slide 1
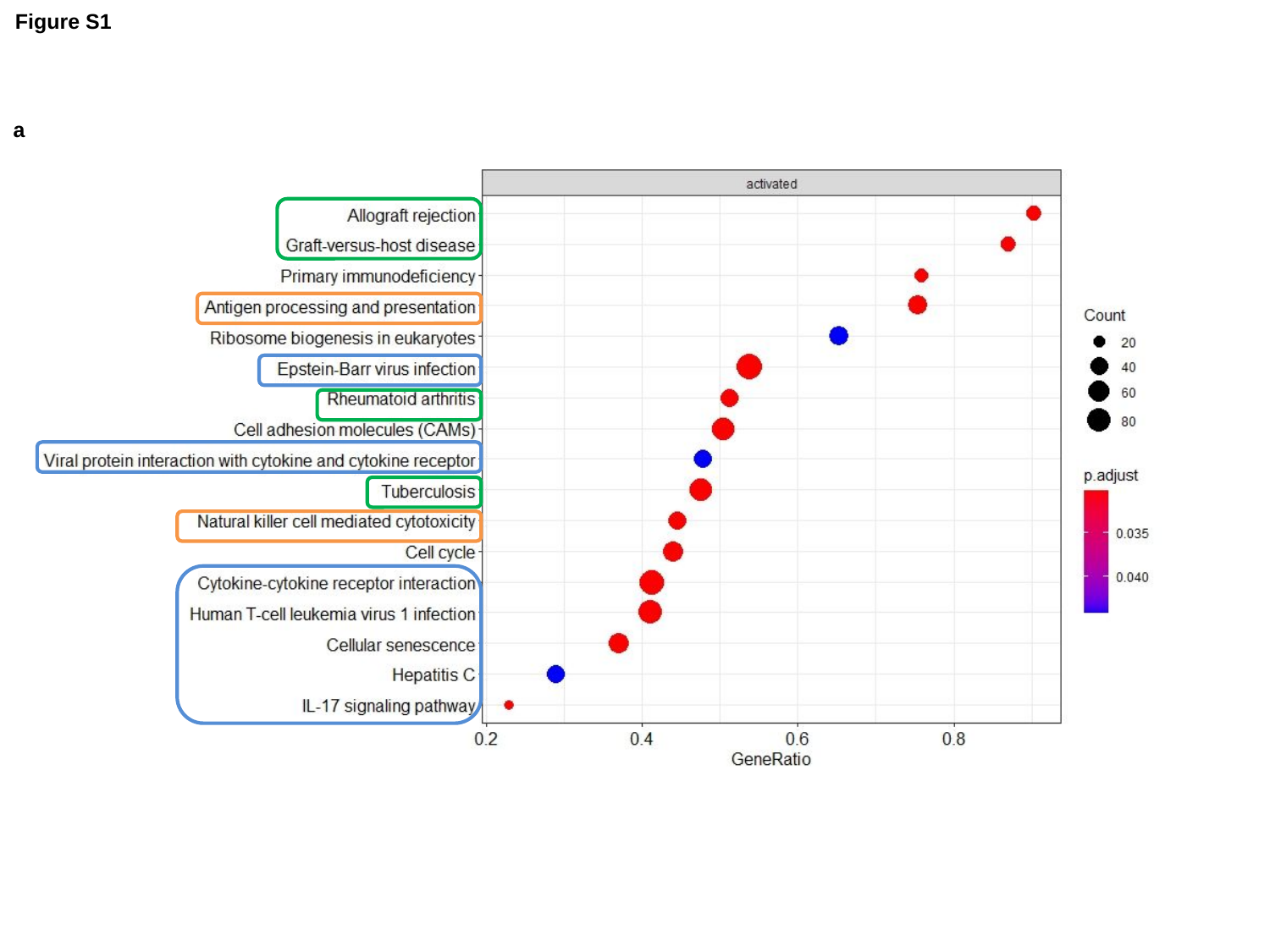

Figure S1
a

## Slide 2
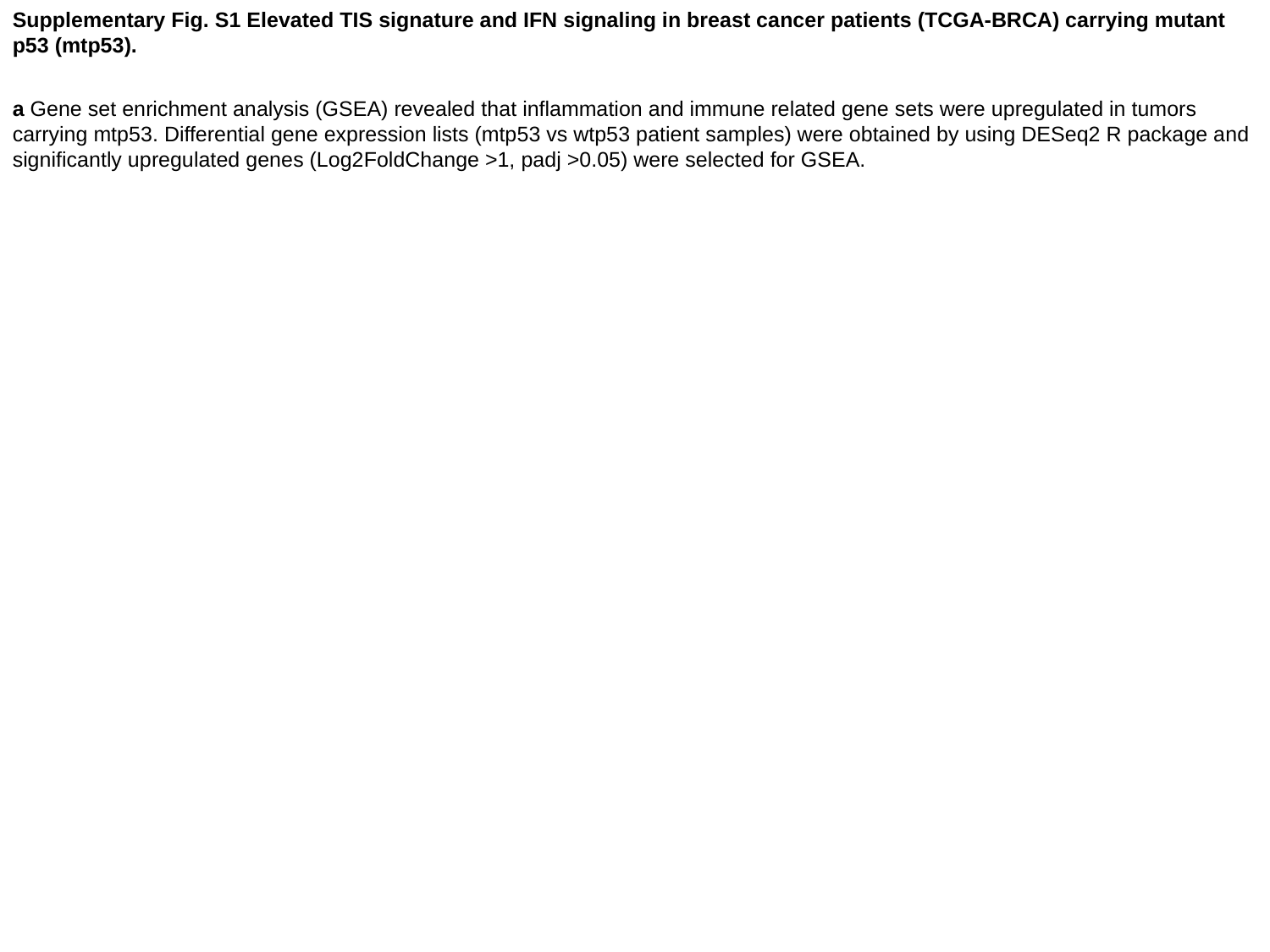

Supplementary Fig. S1 Elevated TIS signature and IFN signaling in breast cancer patients (TCGA-BRCA) carrying mutant p53 (mtp53).
a Gene set enrichment analysis (GSEA) revealed that inflammation and immune related gene sets were upregulated in tumors carrying mtp53. Differential gene expression lists (mtp53 vs wtp53 patient samples) were obtained by using DESeq2 R package and significantly upregulated genes (Log2FoldChange >1, padj >0.05) were selected for GSEA.

## Slide 3
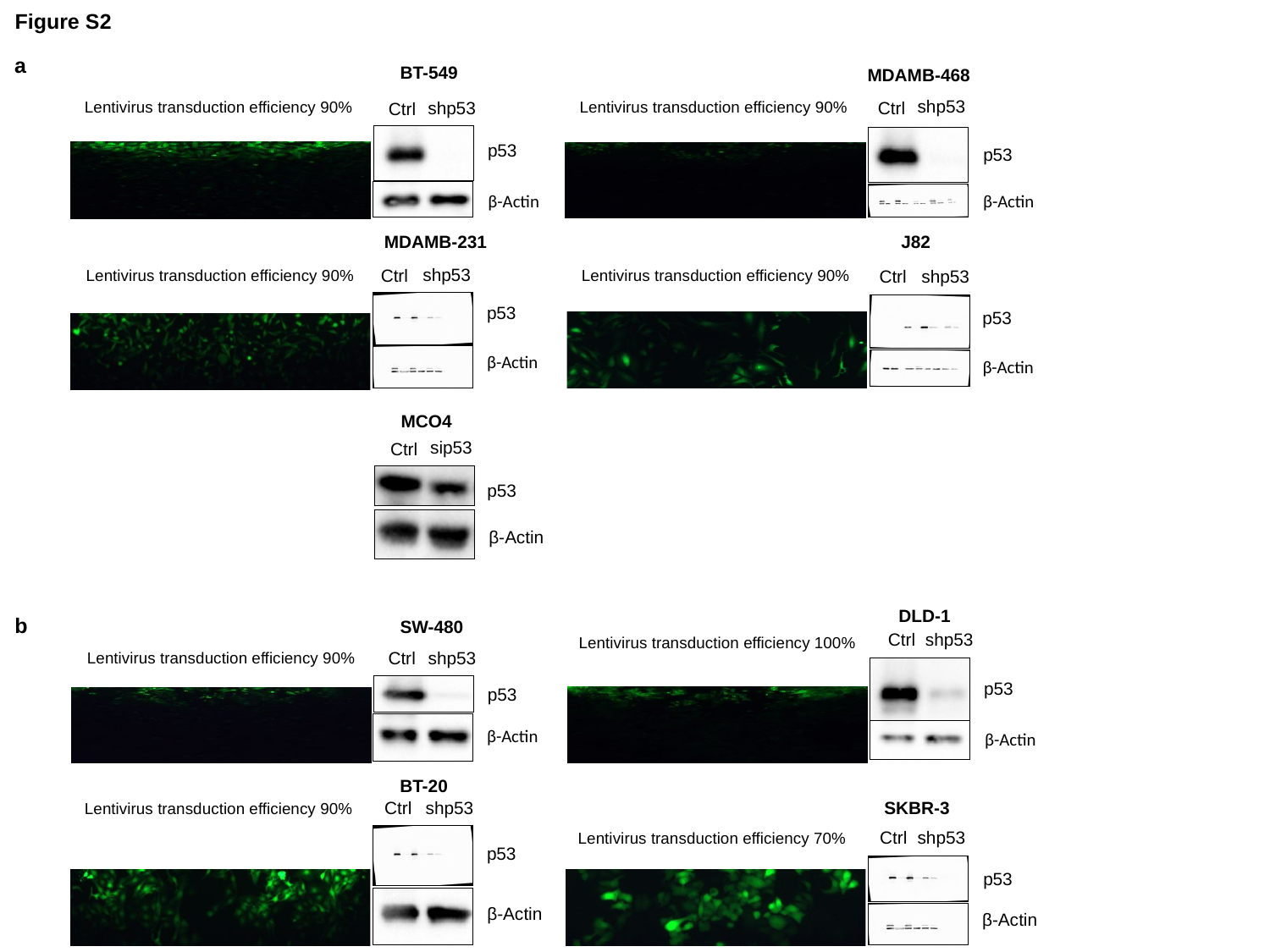

Figure S2
a
BT-549
MDAMB-468
shp53
shp53
Ctrl
Lentivirus transduction efficiency 90%
Lentivirus transduction efficiency 90%
Ctrl
p53
p53
β-Actin
β-Actin
J82
MDAMB-231
shp53
Ctrl
Ctrl
shp53
Lentivirus transduction efficiency 90%
Lentivirus transduction efficiency 90%
p53
p53
β-Actin
β-Actin
MCO4
sip53
Ctrl
p53
β-Actin
DLD-1
b
SW-480
Ctrl
shp53
Lentivirus transduction efficiency 100%
Ctrl
shp53
Lentivirus transduction efficiency 90%
p53
p53
β-Actin
β-Actin
BT-20
SKBR-3
Ctrl
shp53
Lentivirus transduction efficiency 90%
Ctrl
shp53
Lentivirus transduction efficiency 70%
p53
p53
β-Actin
β-Actin

## Slide 4
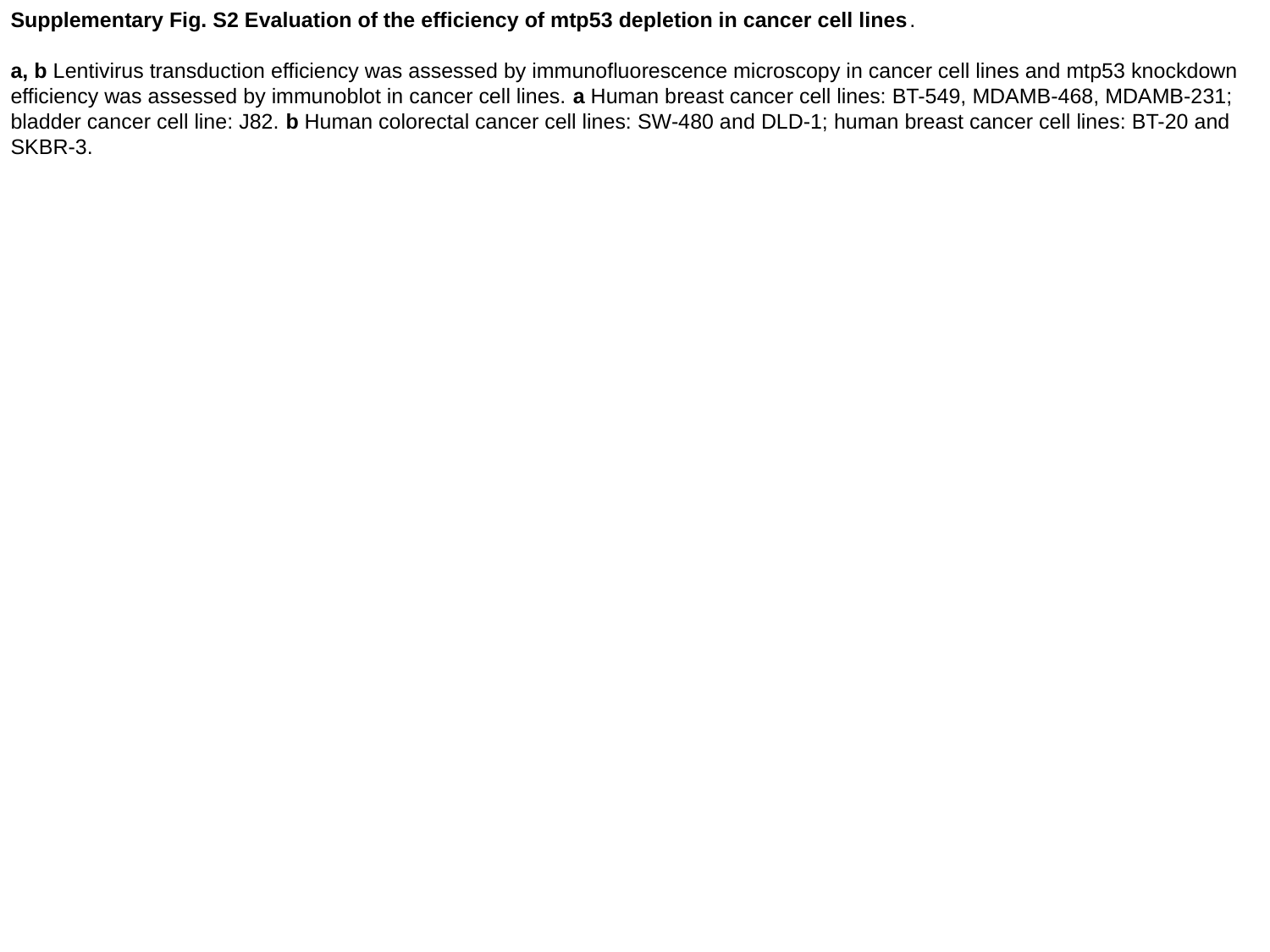

Supplementary Fig. S2 Evaluation of the efficiency of mtp53 depletion in cancer cell lines.
a, b Lentivirus transduction efficiency was assessed by immunofluorescence microscopy in cancer cell lines and mtp53 knockdown efficiency was assessed by immunoblot in cancer cell lines. a Human breast cancer cell lines: BT-549, MDAMB-468, MDAMB-231; bladder cancer cell line: J82. b Human colorectal cancer cell lines: SW-480 and DLD-1; human breast cancer cell lines: BT-20 and SKBR-3.

## Slide 5
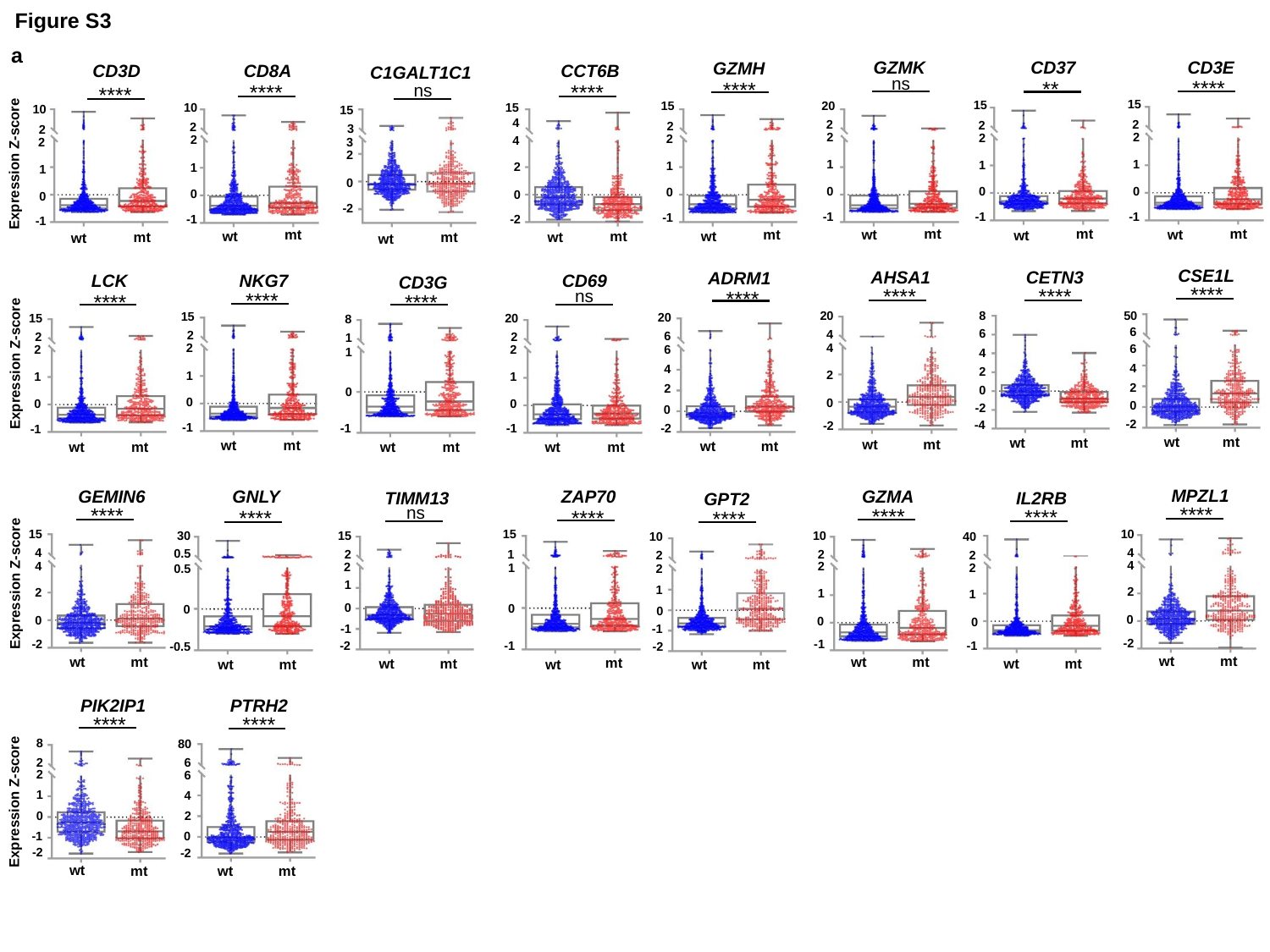

Figure S3
a
GZMK
CD3E
CD37
GZMH
CD3D
CD8A
CCT6B
C1GALT1C1
ns
****
**
****
****
ns
****
****
15
15
15
20
10
15
10
15
Expression Z-score
4
2
2
2
2
2
3
2
2
2
2
2
2
4
3
2
2
1
1
1
1
2
1
1
0
0
0
0
0
0
0
0
-2
-1
-1
-1
-1
-2
-1
-1
mt
mt
mt
wt
wt
mt
mt
wt
wt
mt
wt
mt
wt
mt
wt
wt
CSE1L
CETN3
AHSA1
ADRM1
NKG7
CD69
LCK
CD3G
****
****
****
ns
****
****
****
****
Expression Z-score
20
8
50
15
20
20
15
8
6
6
4
2
6
2
2
1
2
4
6
2
6
2
1
4
4
4
2
2
1
1
1
2
2
0
0
0
0
0
0
0
-2
0
-2
-4
-2
-1
-2
-1
-1
-1
wt
mt
wt
mt
wt
mt
wt
mt
wt
mt
wt
wt
mt
mt
wt
mt
MPZL1
GNLY
ZAP70
GEMIN6
GZMA
TIMM13
IL2RB
GPT2
ns
****
****
****
****
****
****
****
Expression Z-score
15
15
10
15
10
30
10
40
4
4
0.5
1
2
2
2
2
4
4
2
2
2
1
0.5
2
1
1
2
2
1
1
0
0
0
0
0
0
0
0
-1
-1
-2
-2
-1
-2
-1
-1
-0.5
-2
wt
mt
wt
mt
wt
mt
mt
wt
wt
mt
mt
wt
wt
mt
wt
mt
PIK2IP1
PTRH2
****
****
8
80
Expression Z-score
2
6
2
6
1
4
0
2
-1
0
-2
-2
wt
mt
wt
mt

## Slide 6
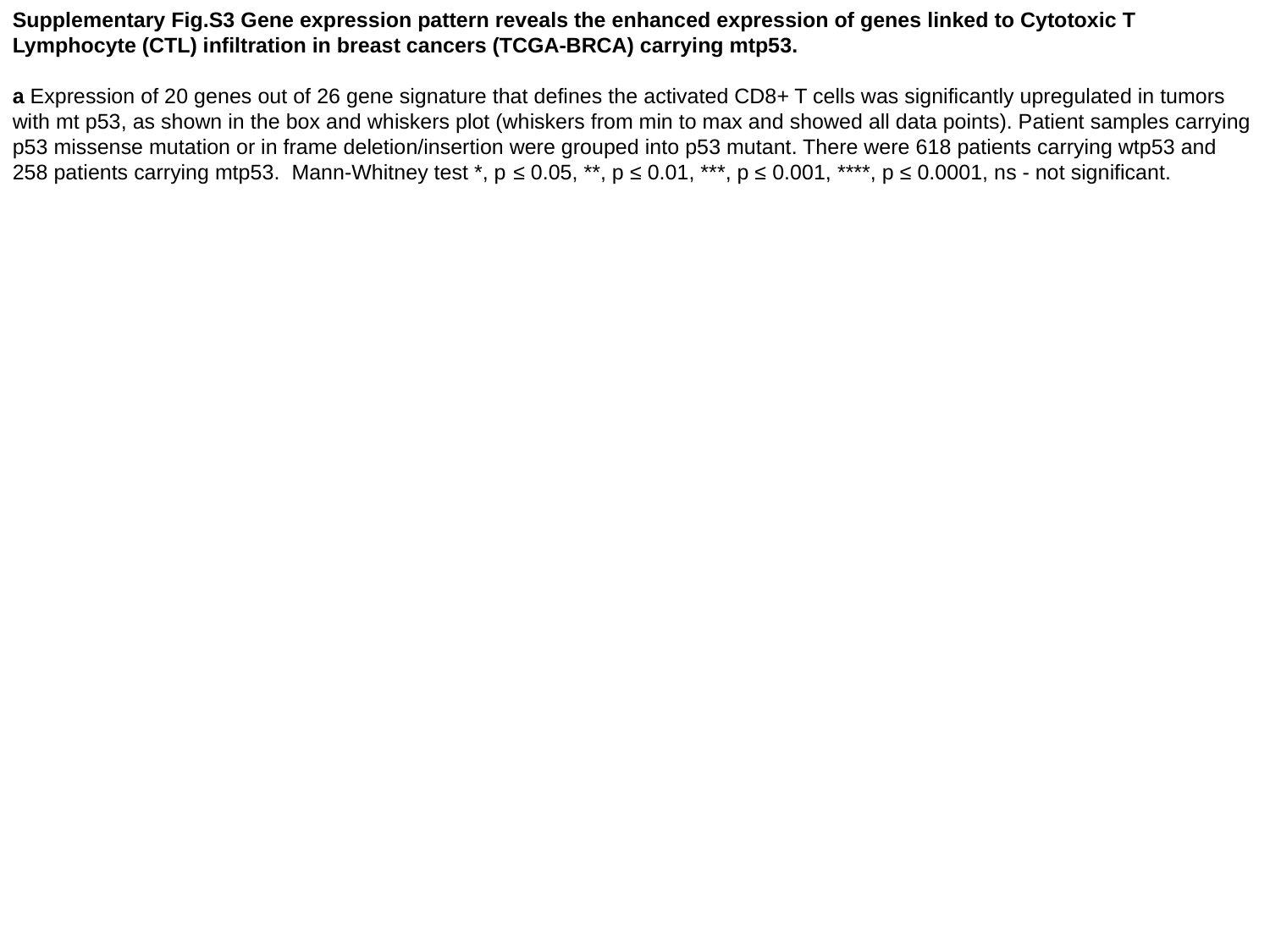

Supplementary Fig.S3 Gene expression pattern reveals the enhanced expression of genes linked to Cytotoxic T Lymphocyte (CTL) infiltration in breast cancers (TCGA-BRCA) carrying mtp53.
a Expression of 20 genes out of 26 gene signature that defines the activated CD8+ T cells was significantly upregulated in tumors with mt p53, as shown in the box and whiskers plot (whiskers from min to max and showed all data points). Patient samples carrying p53 missense mutation or in frame deletion/insertion were grouped into p53 mutant. There were 618 patients carrying wtp53 and 258 patients carrying mtp53. Mann-Whitney test *, p ≤ 0.05, **, p ≤ 0.01, ***, p ≤ 0.001, ****, p ≤ 0.0001, ns - not significant.

## Slide 7
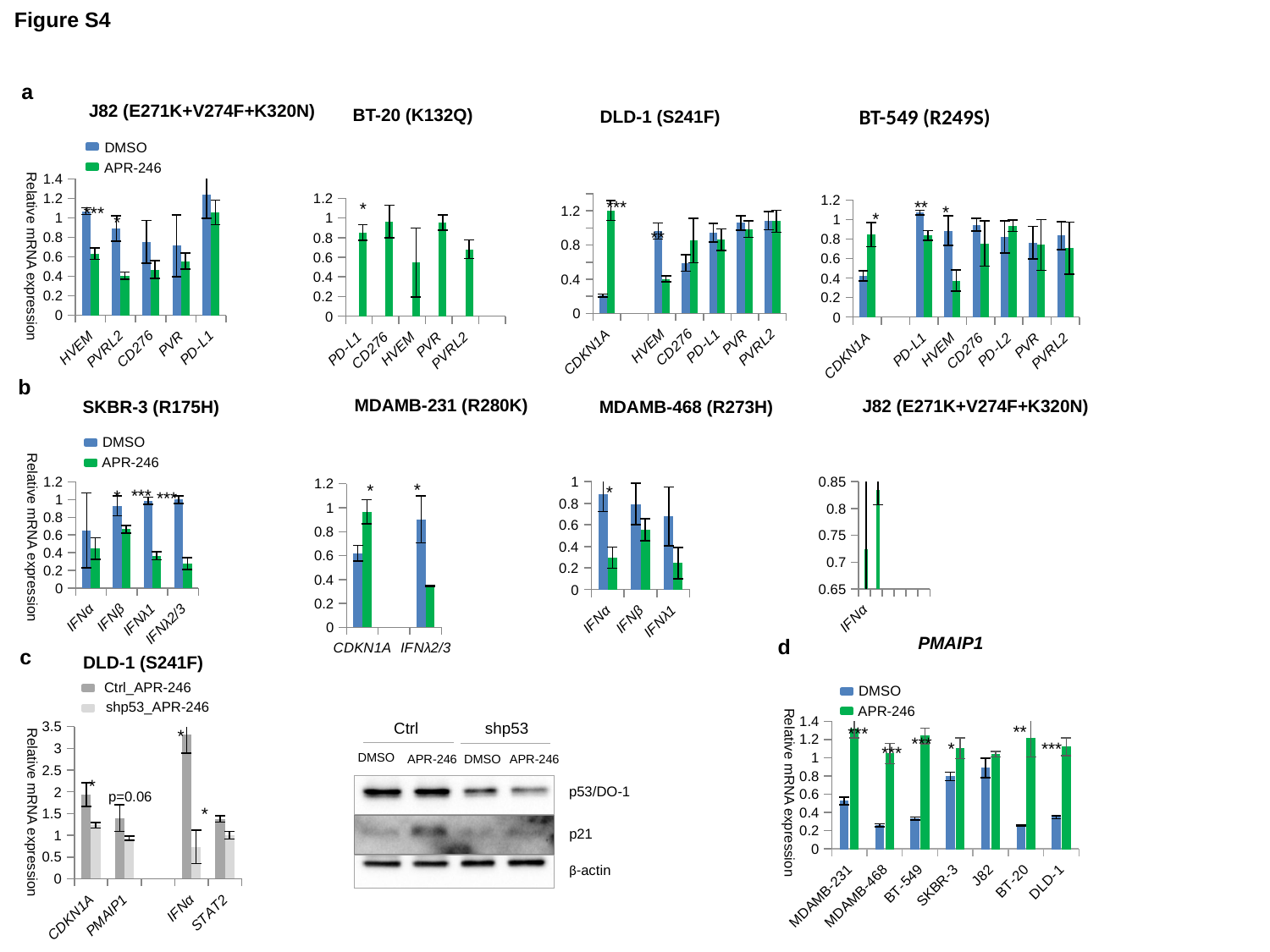

Figure S4
a
J82 (E271K+V274F+K320N)
BT-549 (R249S)
BT-20 (K132Q)
DLD-1 (S241F)
DMSO
APR-246
Relative mRNA expression
### Chart
| Category | | |
|---|---|---|
| HVEM | 0.6326689672475058 | 1.0693533929981967 |
| PVRL2 | 0.40484745840438036 | 0.8912596219437849 |
| CD276 | 0.468170291971691 | 0.7541722814449803 |
| PVR | 0.5562989929541914 | 0.7128768454315164 |
| PD-L1 | 1.0569960026893417 | 1.2355759185318667 |
### Chart
| Category | | |
|---|---|---|
| PD-L1 | 0.851535476397748 | 1.174068727058635 |
| CD276 | 0.963646111073158 | 1.018694213193828 |
| HVEM | 0.545853521074376 | 0.814085907124903 |
| PVR | 0.952844144663579 | 0.60279788870635 |
| PVRL2 | 0.68192761801096 | 0.732628259307913 |
### Chart
| Category | | |
|---|---|---|
| CDKN1A | 0.8485275443937713 | 0.4234885561795317 |
| | None | None |
| PD-L1 | 0.8397330785064664 | 1.0727953975192301 |
| HVEM | 0.3749660756251583 | 0.8878279478989395 |
| CD276 | 0.7563688610412292 | 0.9485706859361486 |
| PD-L2 | 0.9380557252996597 | 0.8235876219315786 |
| PVR | 0.7409317286999997 | 0.7642319728211909 |
| PVRL2 | 0.7086802716286837 | 0.8370811779018217 |**
### Chart
| Category | | |
|---|---|---|
| CDKN1A | 1.2032641383978266 | 0.20959562339852433 |
| | None | None |
| HVEM | 0.40284771157268334 | 0.9637317120371484 |
| CD276 | 0.852707230827674 | 0.5898145276230856 |
| PD-L1 | 0.8623776953769876 | 0.9437985996119403 |
| PVR | 0.984787564433432 | 1.0581470577691907 |
| PVRL2 | 1.0768202592871767 | 1.0846033792409278 |***
*
*
***
*
*
**
b
MDAMB-231 (R280K)
J82 (E271K+V274F+K320N)
SKBR-3 (R175H)
MDAMB-468 (R273H)
DMSO
Relative mRNA expression
APR-246
### Chart
| Category | | |
|---|---|---|
| IFNα | 0.44534698519418 | 0.651238586500053 |
| IFNβ | 0.665234932601701 | 0.928166483064756 |
| IFNλ1 | 0.365270618419701 | 0.986934825271333 |
| IFNλ2/3 | 0.277361745105579 | 1.000332714447945 |
### Chart
| Category | | |
|---|---|---|
| IFNα | 0.296774915032581 | 0.886556105503365 |
| IFNβ | 0.553683628372934 | 0.793592704808125 |
| IFNλ1 | 0.245023386178421 | 0.677996005466199 |
### Chart
| Category | | |
|---|---|---|
| IFNα | 0.72353963413778 | 0.451787926120272 |
| IFNβ | 0.834670817995998 | 1.035079460803141 |
### Chart
| Category | | |
|---|---|---|
| CDKN1A | 0.9650488128350726 | 0.6186113820240303 |
| | None | None |
| IFNλ2/3 | 0.344824034231711 | 0.901772974948892 |*
*
*
***
*
***
PMAIP1
d
c
DLD-1 (S241F)
Ctrl_APR-246
DMSO
shp53_APR-246
APR-246
Relative mRNA expression
### Chart
| Category | | |
|---|---|---|
| MDAMB-231 | 0.525154120486775 | 1.3171279421667001 |
| MDAMB-468 | 0.259270473480483 | 1.0458496978600278 |
| BT-549 | 0.33221754441960066 | 1.2365549040773367 |
| SKBR-3 | 0.7947508264904602 | 1.1038040391552177 |
| J82 | 0.8878343889120416 | 1.03944947471872 |
| BT-20 | 0.25270719833596567 | 1.2086938797544169 |
| DLD-1 | 0.34679213103089196 | 1.1193758732536765 |
Ctrl
shp53
### Chart
| Category | | |
|---|---|---|
| CDKN1A | 1.2327848916577766 | 1.9396967636493 |
| PMAIP1 | 0.9299210079520925 | 1.3955871885878057 |
| | None | None |
| IFNα | 0.7372509962704883 | 3.3210208492971645 |
| STAT2 | 1.0000000000000002 | 1.3780655985930834 |**
Relative mRNA expression
***
*
***
*
***
***
DMSO
APR-246
DMSO
APR-246
*
p53/DO-1
p=0.06
*
p21
β-actin

## Slide 8
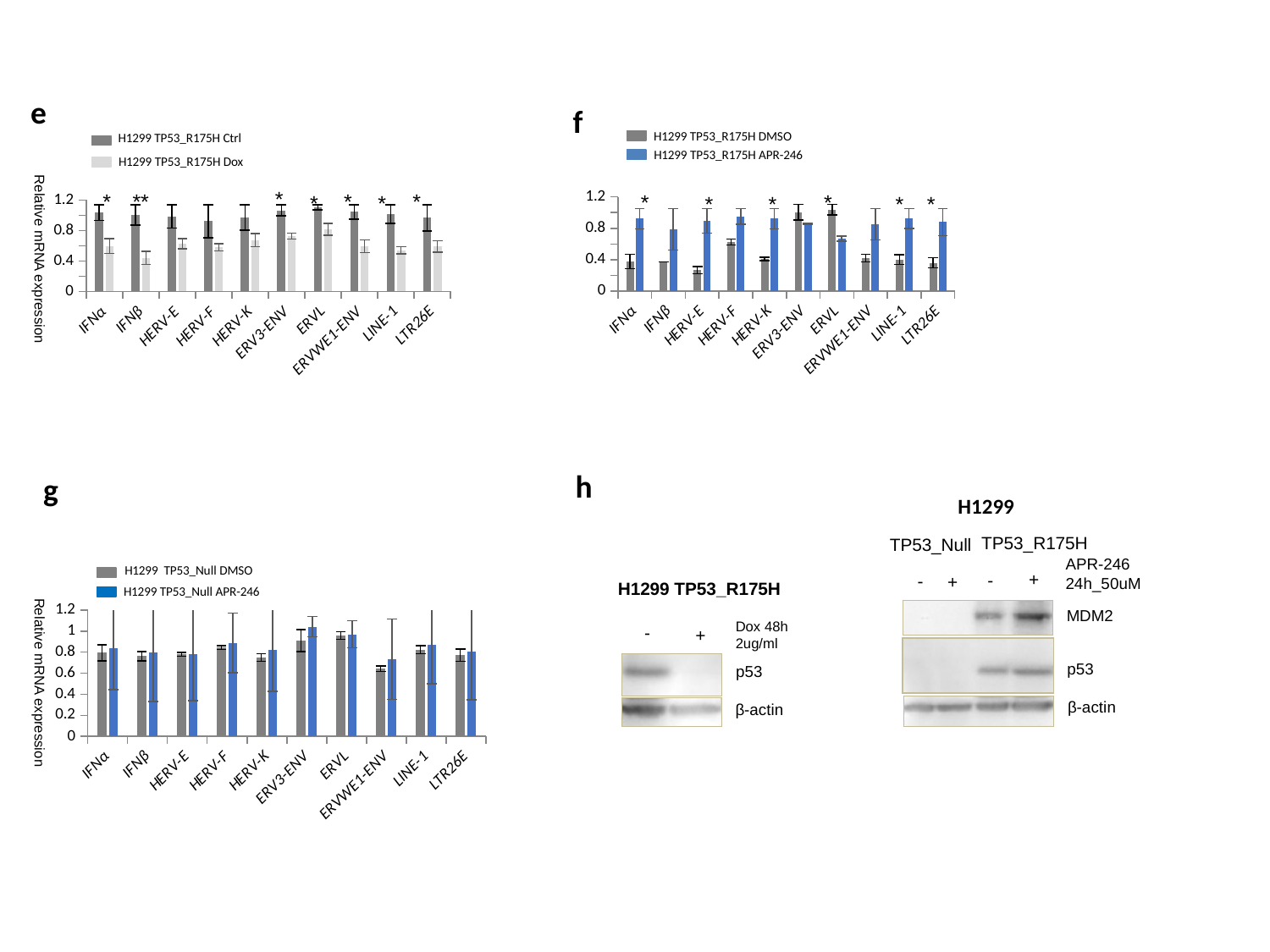

e
f
H1299 TP53_R175H DMSO
H1299 TP53_R175H Ctrl
H1299 TP53_R175H APR-246
H1299 TP53_R175H Dox
Relative mRNA expression
*
*
**
*
*
*
*
*
*
*
*
*
### Chart
| Category | | |
|---|---|---|
| IFNα | 0.3792308672564139 | 0.921670858990792 |
| IFNβ | 0.3709151276690501 | 0.7871218899997678 |
| HERV-E | 0.26819701197246304 | 0.8956035210711317 |
| HERV-F | 0.6245263861859857 | 0.9508210900980449 |
| HERV-K | 0.41078112914849046 | 0.9218517916480986 |
| ERV3-ENV | 1.0041786185473016 | 0.8608164226045014 |
| ERVL | 1.037804301451819 | 0.6673679363981713 |
| ERVWE1-ENV | 0.4205415023835892 | 0.8513463953340967 |
| LINE-1 | 0.40379757335352007 | 0.9228698661007305 |
| LTR26E | 0.3622996129109266 | 0.8780827347037365 |*
### Chart
| Category | | |
|---|---|---|
| IFNα | 1.034393185901244 | 0.5939605844560637 |
| IFNβ | 1.0035568523883762 | 0.44048438546744917 |
| HERV-E | 0.9842517540638434 | 0.6264756953273974 |
| HERV-F | 0.9219797928926704 | 0.5788400252534236 |
| HERV-K | 0.9707268839333871 | 0.6739114239703122 |
| ERV3-ENV | 1.0646945545100936 | 0.7264507153441252 |
| ERVL | 1.1036846405247371 | 0.8144116913865705 |
| ERVWE1-ENV | 1.0438467972418168 | 0.5919917591084852 |
| LINE-1 | 1.0155193618199918 | 0.5407534756626116 |
| LTR26E | 0.9656996604966696 | 0.5920181359316397 |h
g
H1299
TP53_R175H
TP53_Null
APR-246
24h_50uM
H1299 TP53_Null DMSO
+
-
-
+
H1299 TP53_R175H
H1299 TP53_Null APR-246
Relative mRNA expression
### Chart
| Category | | |
|---|---|---|
| IFNα | 0.7939962450021533 | 0.8382244283873387 |
| IFNβ | 0.7616510855152807 | 0.7942266538470127 |
| HERV-E | 0.7793074407665118 | 0.7833644459533851 |
| HERV-F | 0.842711507735426 | 0.8869873247453759 |
| HERV-K | 0.7483450826627905 | 0.823283139978478 |
| ERV3-ENV | 0.9095810371218196 | 1.0414332272566138 |
| ERVL | 0.958306979353829 | 0.9691600622814075 |
| ERVWE1-ENV | 0.642850716410908 | 0.7320089207035313 |
| LINE-1 | 0.8234574949672316 | 0.8659456596089967 |
| LTR26E | 0.7701679278504868 | 0.8030105514746008 |MDM2
Dox 48h
2ug/ml
-
+
p53
p53
β-actin
β-actin

## Slide 9
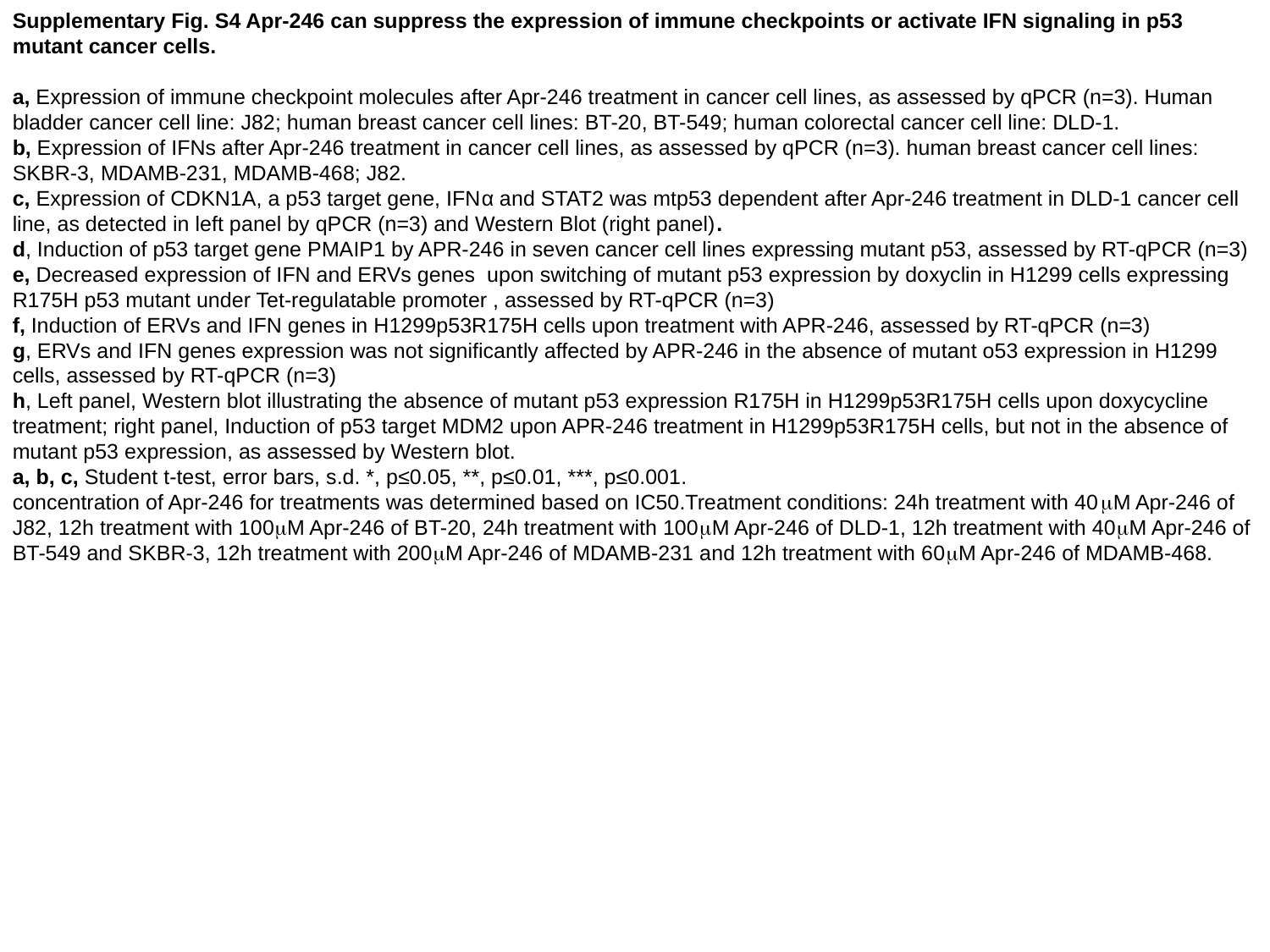

Supplementary Fig. S4 Apr-246 can suppress the expression of immune checkpoints or activate IFN signaling in p53 mutant cancer cells.
a, Expression of immune checkpoint molecules after Apr-246 treatment in cancer cell lines, as assessed by qPCR (n=3). Human bladder cancer cell line: J82; human breast cancer cell lines: BT-20, BT-549; human colorectal cancer cell line: DLD-1.
b, Expression of IFNs after Apr-246 treatment in cancer cell lines, as assessed by qPCR (n=3). human breast cancer cell lines: SKBR-3, MDAMB-231, MDAMB-468; J82.
c, Expression of CDKN1A, a p53 target gene, IFNα and STAT2 was mtp53 dependent after Apr-246 treatment in DLD-1 cancer cell line, as detected in left panel by qPCR (n=3) and Western Blot (right panel).
d, Induction of p53 target gene PMAIP1 by APR-246 in seven cancer cell lines expressing mutant p53, assessed by RT-qPCR (n=3)
e, Decreased expression of IFN and ERVs genes upon switching of mutant p53 expression by doxyclin in H1299 cells expressing R175H p53 mutant under Tet-regulatable promoter , assessed by RT-qPCR (n=3)
f, Induction of ERVs and IFN genes in H1299p53R175H cells upon treatment with APR-246, assessed by RT-qPCR (n=3)
g, ERVs and IFN genes expression was not significantly affected by APR-246 in the absence of mutant o53 expression in H1299 cells, assessed by RT-qPCR (n=3)
h, Left panel, Western blot illustrating the absence of mutant p53 expression R175H in H1299p53R175H cells upon doxycycline treatment; right panel, Induction of p53 target MDM2 upon APR-246 treatment in H1299p53R175H cells, but not in the absence of mutant p53 expression, as assessed by Western blot.
a, b, c, Student t-test, error bars, s.d. *, p≤0.05, **, p≤0.01, ***, p≤0.001.
concentration of Apr-246 for treatments was determined based on IC50.Treatment conditions: 24h treatment with 40μM Apr-246 of J82, 12h treatment with 100μM Apr-246 of BT-20, 24h treatment with 100μM Apr-246 of DLD-1, 12h treatment with 40μM Apr-246 of BT-549 and SKBR-3, 12h treatment with 200μM Apr-246 of MDAMB-231 and 12h treatment with 60μM Apr-246 of MDAMB-468.

## Slide 10
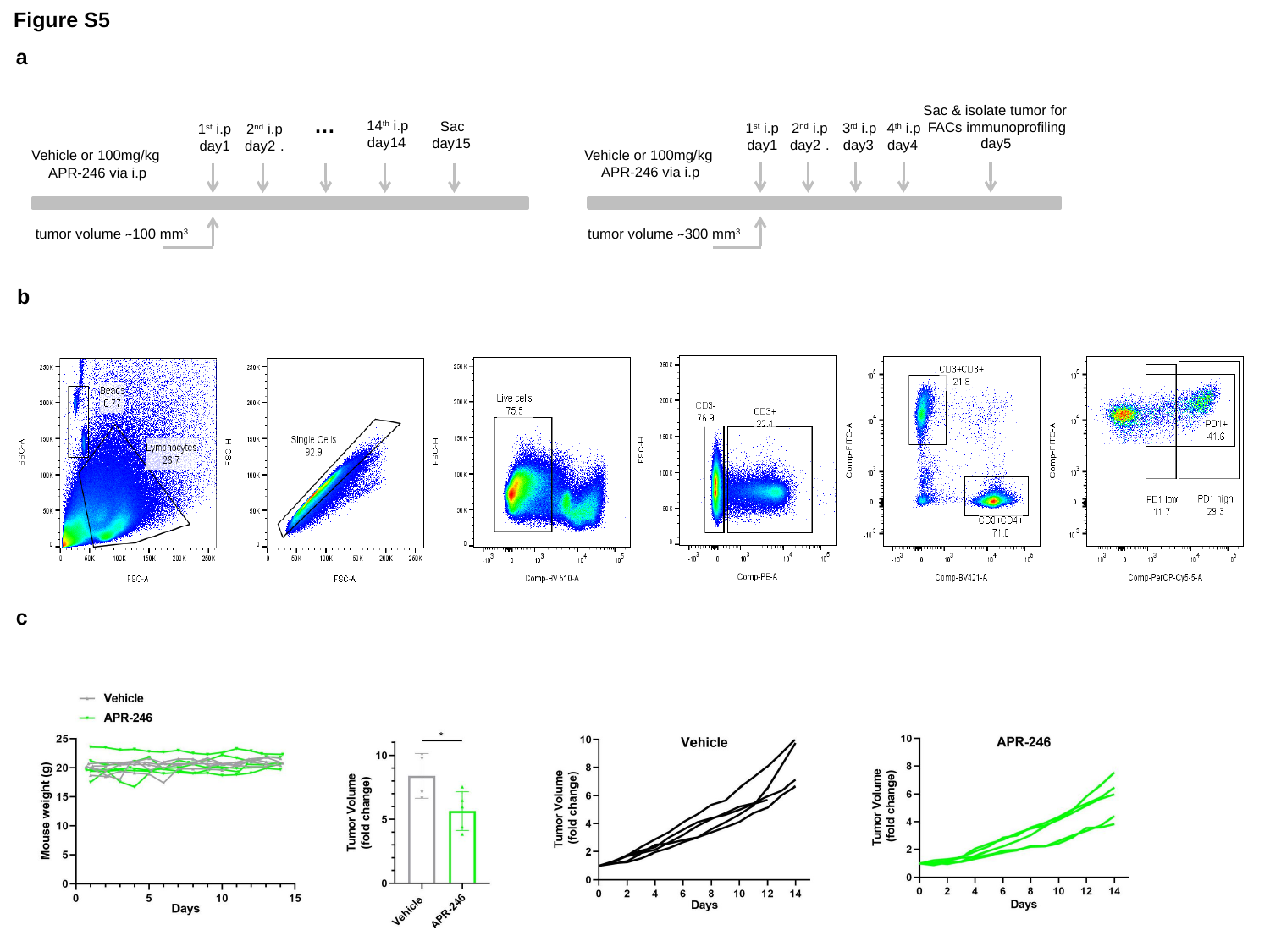

Figure S5
a
Sac & isolate tumor for
 FACs immunoprofiling
 day5
1st i.p
day1
2nd i.p
day2 .
3rd i.p
day3
4th i.p
day4
Vehicle or 100mg/kg
APR-246 via i.p
tumor volume ~300 mm3
…
14th i.p
day14
Sac
day15
1st i.p
day1
2nd i.p
day2 .
Vehicle or 100mg/kg
APR-246 via i.p
tumor volume ~100 mm3
b
c

## Slide 11
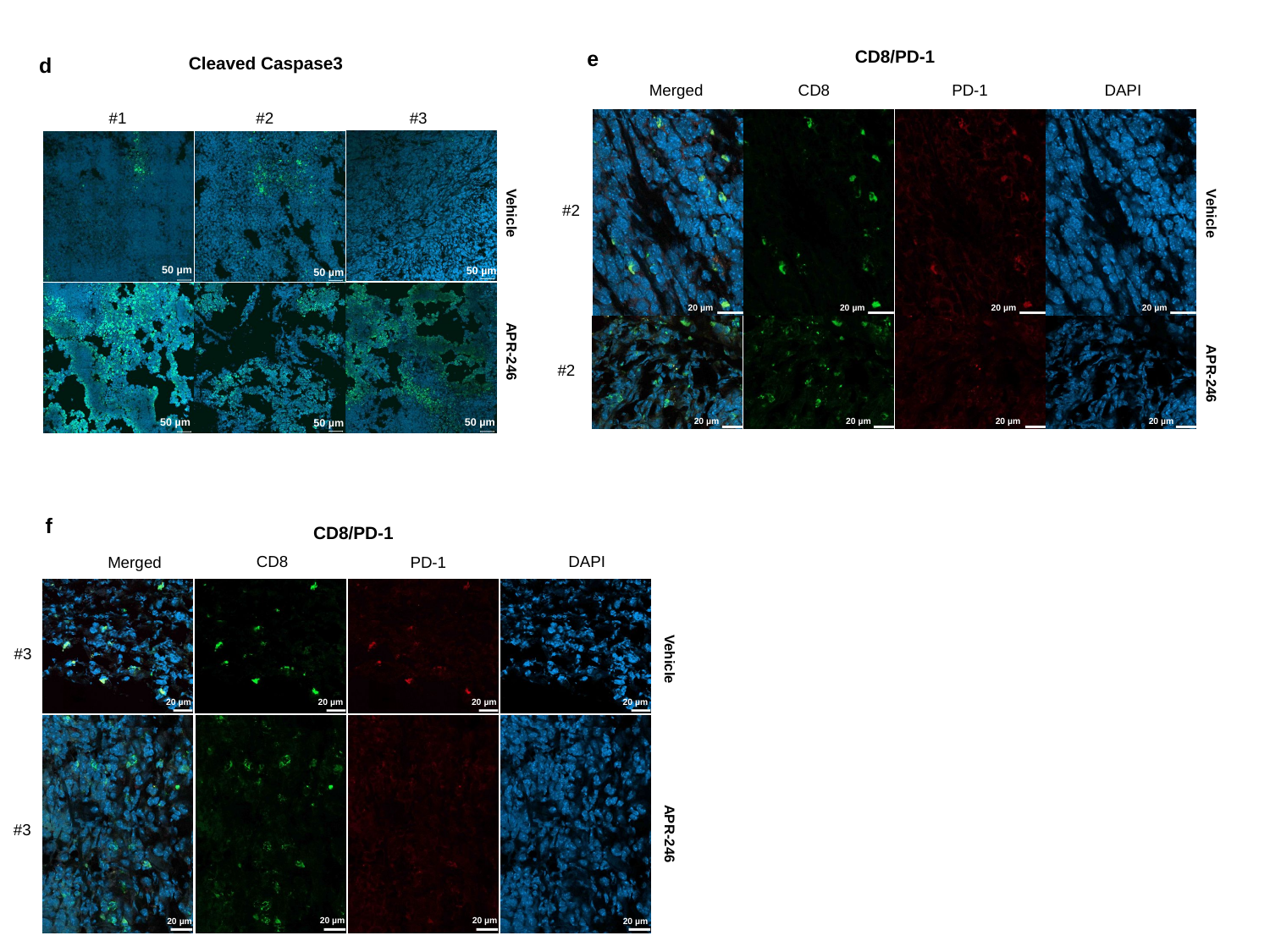

e
CD8/PD-1
d
Cleaved Caspase3
Merged
CD8
PD-1
DAPI
#1
#2
#3
#2
Vehicle
Vehicle
50 µm
50 µm
50 µm
20 µm
20 µm
20 µm
20 µm
APR-246
#2
APR-246
50 µm
50 µm
20 µm
20 µm
20 µm
20 µm
50 µm
f
CD8/PD-1
CD8
DAPI
PD-1
Merged
#1
#3
Vehicle
20 µm
20 µm
20 µm
20 µm
#1
#3
APR-246
20 µm
20 µm
20 µm
20 µm

## Slide 12
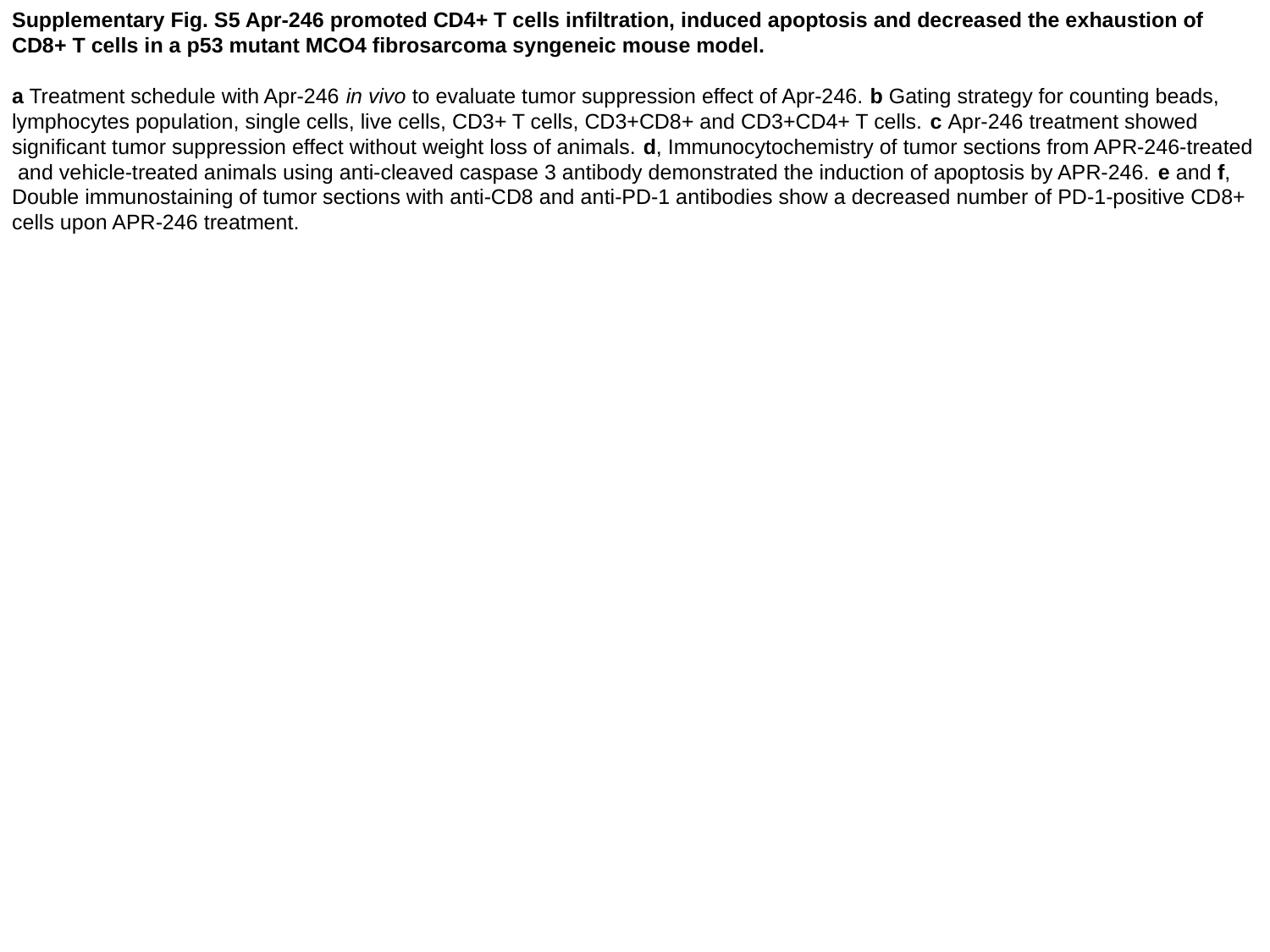

Supplementary Fig. S5 Apr-246 promoted CD4+ T cells infiltration, induced apoptosis and decreased the exhaustion of CD8+ T cells in a p53 mutant MCO4 fibrosarcoma syngeneic mouse model.
a Treatment schedule with Apr-246 in vivo to evaluate tumor suppression effect of Apr-246. b Gating strategy for counting beads, lymphocytes population, single cells, live cells, CD3+ T cells, CD3+CD8+ and CD3+CD4+ T cells. c Apr-246 treatment showed significant tumor suppression effect without weight loss of animals. d, Immunocytochemistry of tumor sections from APR-246-treated and vehicle-treated animals using anti-cleaved caspase 3 antibody demonstrated the induction of apoptosis by APR-246. e and f, Double immunostaining of tumor sections with anti-CD8 and anti-PD-1 antibodies show a decreased number of PD-1-positive CD8+ cells upon APR-246 treatment.

## Slide 13
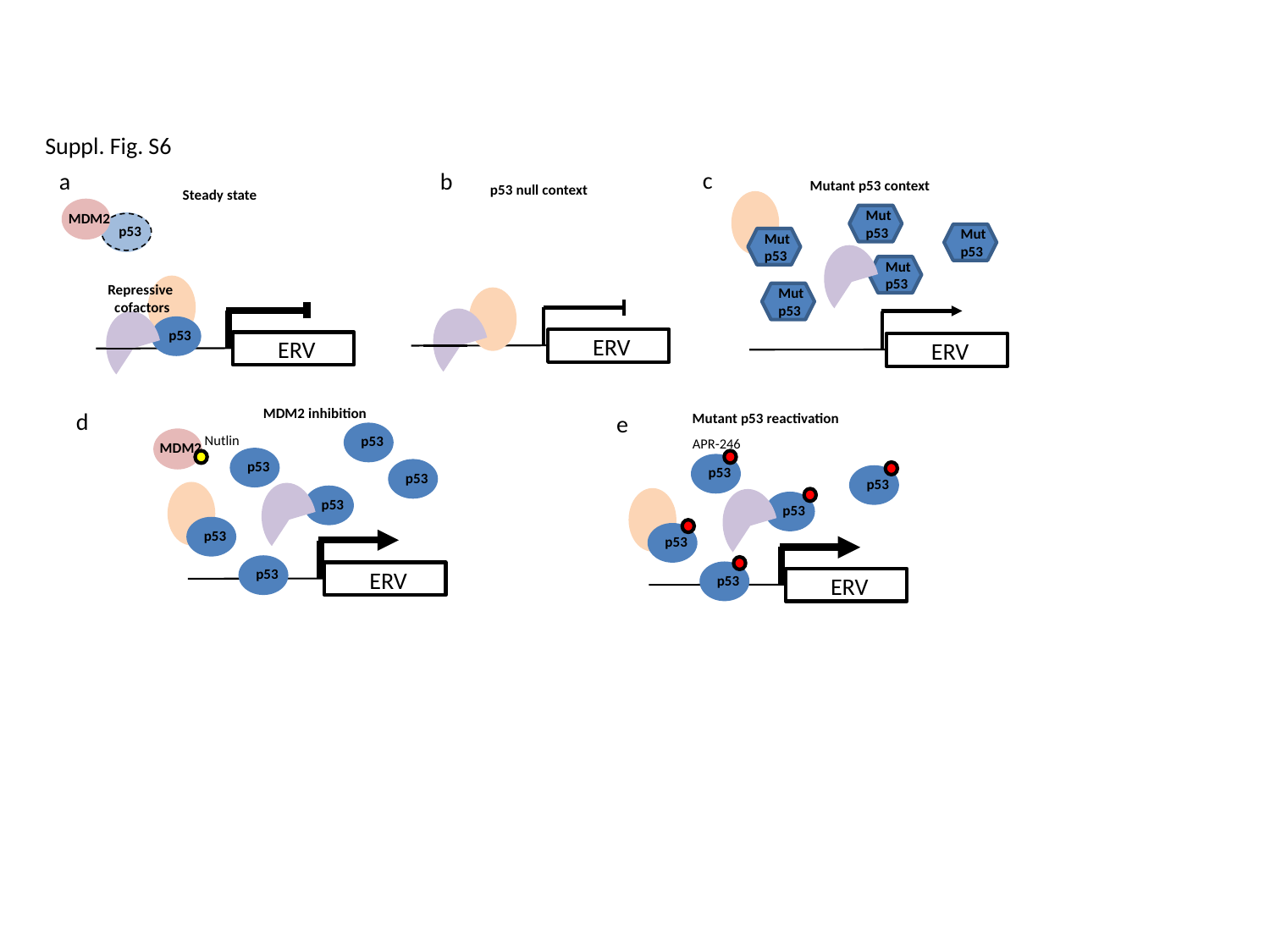

Suppl. Fig. S6
c
a
b
Mutant p53 context
Mut
p53
Mut
p53
Mut
p53
Mut
p53
Mut
p53
ERV
p53 null context
ERV
Steady state
MDM2
p53
Repressive
cofactors
p53
ERV
MDM2 inhibition
p53
Nutlin
MDM2
p53
p53
p53
p53
p53
ERV
d
e
Mutant p53 reactivation
APR-246
p53
p53
p53
p53
p53
ERV

## Slide 14
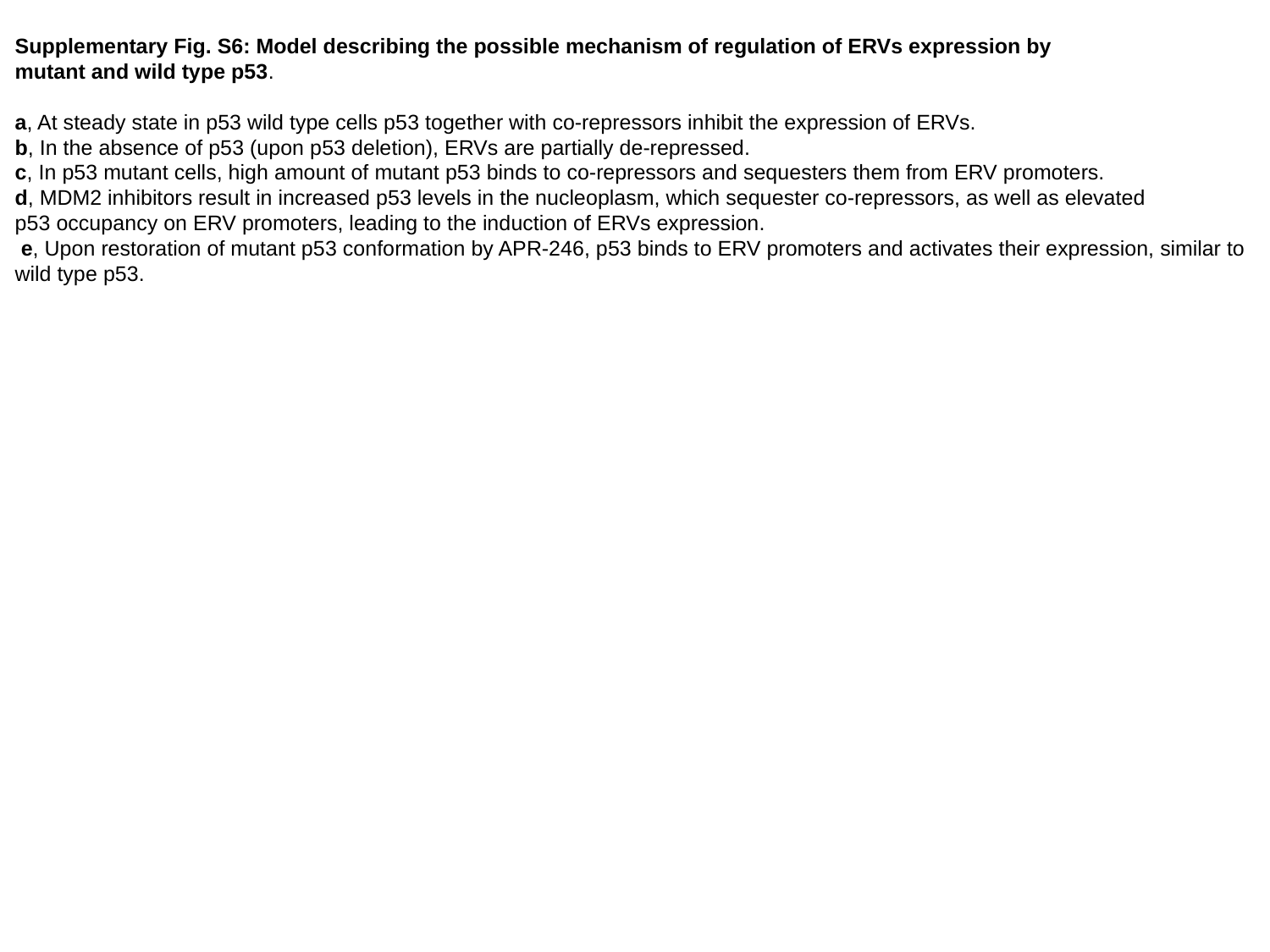

Supplementary Fig. S6: Model describing the possible mechanism of regulation of ERVs expression by
mutant and wild type p53.
a, At steady state in p53 wild type cells p53 together with co-repressors inhibit the expression of ERVs.
b, In the absence of p53 (upon p53 deletion), ERVs are partially de-repressed.
c, In p53 mutant cells, high amount of mutant p53 binds to co-repressors and sequesters them from ERV promoters.
d, MDM2 inhibitors result in increased p53 levels in the nucleoplasm, which sequester co-repressors, as well as elevated
p53 occupancy on ERV promoters, leading to the induction of ERVs expression.
 e, Upon restoration of mutant p53 conformation by APR-246, p53 binds to ERV promoters and activates their expression, similar to
wild type p53.
