# Supplementary material for: Mutant p53 gain of function mediates cancer immune escape that is counteracted by APR-246
Source: Br J Cancer. 2022 Sep 22;127(11):2060–71. doi: 10.1038/s41416-022-01971-8 (PMC9681866; doi:10.1038/s41416-022-01971-8)
Supplement: Supplementary file 2 — Supplementary Method [file 41416_2022_1971_MOESM2_ESM.docx]

**Mutant p53 gain of function induces an inflamed tumor microenvironment that is counteracted by APR-246**

Xiaolei Zhou^1^, Gema Sanz Santos^1^, Yue Zhan^1,2^, Mariana M.S. Oliveira^1^, Shiva Rezaei^1^, Madhurendra Singh^1^, Sylvain Peuget^1^, Lisa S. Westerberg^1^, John Inge Johnsen^3^ and Galina Selivanova^1,4^

^1^Department of Microbiology, Tumor and Cell Biology, Karolinska Institutet, Stockholm, 171 65, Sweden

^2^Department of Breast Surgery, The First Hospital of Jilin University, Changchun, China,

^3^Department of Women's and Children's Health, Childhood Cancer Research Unit, Karolinska Institutet, Stockholm, 171 77 Sweden

^4^Corresponding author: Galina Selivanova, galina.selivanova@ki.se; Department of Microbiology, Tumor and Cell Biology, Biomedicum C8, Karolinska Institutet, 171 65 Stockholm; Sweden; Tel: +46 8 52486302.

**Methods**

**Immunoprofiling of tumor samples by flow cytometry**

Isolated tumor samples were mechanically disrupted by medical blade and syringe plunger, filtered through a 70 μm cell strainer for two times. After centrifugation at 300g for 5 mins, cell pellet was collected and dissolved in 5 ml PBS. Lymphocytes were separated from tumor cells by Ficoll separation. Briefly, 5 ml of PBS solution containing the tumor cells was overlaid on 5 ml of Ficoll Paque Plus (Cytiva, cat #17-1440-02), centrifuged at 2000 rpm for 20 min with acceleration 3 and no brake. Tumor infiltrating lymphocytes were collected, stained and analyzed by flow cytometer, LSRFortessa X-20 (BD Biosciences). All surface staining was performed in phosphate-buffered saline (PBS) with1% FBS after blocking unspecific staining via FcgRIII/II with purified anti-CD16/32 (BD Pharmingen; clone 2.4G2; cat#553142). Dead cells were excluded from gating with the Live/Dead Fixable Aqua Dead Cell Stain (Invitrogen; L34966). The antibodies used for flow cytometry were as follows: CD3-PE (Biolegend; clone 145-2C11; cat#100308), CD4-PB (Invitrogen; clone RM4-5; cat#MCD0428), CD8-FITC (Biolegend; clone 53-6.7; cat#100706), CD11b-AF700 (Biolegend; clone M1/70; cat#101222), Nkp46-AF647 (Biolegend; clone 29A1.4; cat#137628), NKG2D-PECy7 (Invitrogen; clone CX5; cat#25-5882-82), CD69-PECy5 (Biolegend; clone H1.2F3, cat#104510), KLRG1-bio (BD Pharmingen; clone 2F1; cat#550863), SA-QD605 (Invitrogen; cat#Q10101MP), PD1-PerCPeFluor710 (Invitrogen; clone RMP1-30; cat#46-9981-82), CD3-AF700 (Biolegend, clone 17A2, cat#100216), CTLA4-APC (Biolegend; clone UC10-4B9; cat#106309), FoxP3-PE (eBioscience; clone FJK-16s; cat#12-5773-82), IFNγ-PerCpCy5.5 (Biolegend; clone XMG1.2; cat#505821). Data were analyzed and figures were prepared by Flowjo (version 10.5.3 Tree Star Inc) and GraphPad Prism 5, respectively.

**qPCR running program and data analysis**

The following program was used in qPCR machine: 95 ℃ for 2 minutes; 40 cycles from 90 ℃ for 10 seconds, to 60 ℃ for 45 seconds followed by plate read; Melt Curve was measured from 65 ℃ to 95 ℃ with 0.5 ℃ increment for 5 seconds and followed by plate read. RT-qPCR data was analyzed by 2^(-ΔΔCq) method with software Bio-Rad CFX Maestro (version: 4.1.2433.1219) and multiple control genes were used including β-Actin, GAPDH and RPLP0. Amplification efficiency and specificity of primers were tested before use. Primers sequences can be found in Supplementary Table S2.

**Calculation of gene ratio related to Fig S1a and Z-score**

Gene ratio is the ratio of input genes, in our case of differentially expressed genes (DEGs) that are annotated in a term or gene set. Gene ratio is calculated by the following formula: (number of DEGs that belong to a given gene set) / (total number of genes in a given gene set). A low or a high gene ratio of a particular gene set means lower or higher number of DEGs presented in a given gene set.

Z-score transform the data by centering and scaling each genes’ expression by their mean and standard deviation, respectively. Z-score can be interpreted as the number of standard deviations of a gene expression away from the mean of the gene expression across all samples. A positive z-score means that the gene’s expression was above the average value across samples, whereas a negative z-score means it was below the average. The gene expression was exactly average if z-score equal to zero.

**Detailed protocol of Western blot**

After transferring proteins from gels to membrane, 5% fat-free milk in PBST was used to block the membrane for 1h at room temperature (RT) followed by incubation of primary antibody to detect the target protein overnight at 4 ℃. After washing with 3 times PBST, membrane was incubated with HRP-conjugated secondary antibody for 1h at RT and subsequently washed with 3 times PBST. HRP-substrate (SuperSignal^TM^ West Dura Extended Duration Substrate detection system, Cat# 34075, Thermo Fisher Scientific) was applied to the blotted protein membrane and colorimetric detection was performed by using ChemiDocTM XRS+ Imaging System (Bio-Rad). Protein bands on the membrane were quantified by Image J software if required. The antibodies used for Western Blot were as follows: p53 (Clone DO-1, cat# sc-126, Santa Cruz Biotechnology) for detection of human p53, β-actin (Clone C4, Cat# MAB1501, Millipore), p53 (Clone A-1, cat# sc-393031, Santa Cruz Biotechnology) for detection of murine p53 and MDM2 (Clone IF2, Cat# 182403, Thermo Fisher Scientific).

**Cell culture**

Human breast cancer BT-549, human colorectal cancer DLD-1, human non-small cell lung cancer H1299 TP53_null and H1299 TP53_R175H cells lines were cultured in RPMI-1640 medium (Hyclone) supplemented with 10% FBS (Hyclone), 100 U/ml of penicillin and 100 mg/ml of streptomycin (Sigma-Aldrich). Human breast cancer MDAMB-231, MDAMB-468 and SKBR-3, human colorectal cancer SW480, HCT116 TP53_wildtype, HCT116 TP53_R248W and HCT116 TP53_knockout, murine fibrosarcoma MCO4 cancer cells were cultured in DMEM medium (Hyclone) supplemented with 10% FBS, and antibiotics. Human breast cancer BT-20 and human urinary bladder cancer cell lines were cultured in EMEM medium (Hyclone) supplemented with 10% FBS, and antibiotics. All cells were purchased from ATCC in recent years and only the early passaged cells (less than 10 times) were used. All cell lines were incubated in 5% CO2 incubator at 37°C and tested negative for mycoplasma before assay.

**Compounds**

Mutant p53-activating compound APR-246 (provided by Aprea Therapeutics) was reconstituted in DMSO (100 mmol/L) for *in vitro* use and was reconstituted in PBS (50mg/ml) for i.p. injection in mouse experiment. Nutlin-3 (purchased from Sigma-Aldrich) was reconstituted in DMSO (10 mmol/L). Doxycycline (purchased from Sigma-Aldrich) was reconstituted in nuclease free water. The treatment concentration and time of APR-246, Nutlin-3 and Doxycycline on different cancer cell lines were mentioned in the corresponding figure legends.

**Immunohistochemistry (IHC) detection of ISG15, cleaved-caspase3, CD8 and PD-1 on BALB/c Mouse Model**

Mice were euthanized by CO2 inhalation 24h after the final treatment either with vehicle or APR-246. Then tumor samples were isolated, washed with PBS and immediately mounted by optimal cutting temperature compound (OCT compound, Cat# 361603E, VWR) under dry ice. Tumor sample slices (10μm) have been cut by NX70 cryostats at the Histological Core Facility of Karolinska Institutet and dried onto adhesion glass slide (Cat# 10422755, Thermo Fisher Scientific). Next, tissue slices were fixed in pre-chilled 4% PFA at room temperature (RT) for 30 mins in tank followed by 3 times wash by PBS. Tissue slices were blocked by blocking buffer (0.1% TritonX-100, 5% donkey serum in PBS) for 1h at RT followed by primary antibody (at 1.100 dilution in blocking buffer otherwise stated) incubation at 4°C overnight in a humified chamber, including either anti-ISG15 alone (Clone F-9, Cat# sc-166755, Santa Cruz Biotechnology), or co-staining with anti- CD8 (FITC-anti-mouse CD8a, Clone 53-6.7, Cat# 100706, BioLegend)fa and PD-1 (APC-anti-mouse CD279/PD-1, Clone RMP1-30, Cat# 109112, BioLegend), or anti-Cleaved-Caspase 3 alone at 1:200 dilution in blocking buffer (Asp175, Cat# 9661S, CST). After 3 times washing by PBS, AF568 labelled donkey anti-mouse (Cat# A10037, Invitrogen) or AF488 labelled donkey anti-rabbit (Cat# PIA32790TR, Invitrogen) secondary antibody at 1:1000 dilution in blocking buffer was applied to either ISG15 or Cleaved-Caspase3 staining for 1h at RT in dark and then 3 times washing with PBS. Samples were mounted with mounting media Prolong gold (Invitrogen) with 4,6-diamidino-2-phenylindole (DAPI) and 24h later images were taken by Zeiss LSM800-Airy with 6*6 tiles and 5 supporting points.
